# Estimates of case-fatality ratios of measles in low-income and middle-income countries: a systematic review and modelling analysis

**DOI:** 10.1016/S2214-109X(18)30537-0

**Published:** 2019-02-21

**Authors:** Allison Portnoy, Mark Jit, Matthew Ferrari, Matthew Hanson, Logan Brenzel, Stéphane Verguet

**Affiliations:** a Department of Global Health and Population, Harvard T H Chan School of Public Health, Boston, MA, USA; b Department of Infectious Disease Epidemiology, London School of Hygiene and Tropical Medicine, London, UK; c Modelling and Economics Unit, Public Health England, London, UK; d The Center for Infectious Disease Dynamics, Department of Biology and Department of Statistics, The Pennsylvania State University, University Park, PA, USA; e Bill & Melinda Gates Foundation, Seattle, WA, USA

## Abstract

**Background:**

In the 21st century, increases in immunisation coverage and decreases in under-5 mortality have substantially reduced the global burden of measles mortality. However, the assessment of measles mortality burden is highly dependent on estimates of case-fatality ratios for measles, which can vary according to geography, health systems infrastructure, prevalence of underlying risk factors, and measles endemicity. With imprecise case-fatality ratios, there is continued uncertainty about the burden of measles mortality and the effect of measles vaccination. In this study, we aimed to update the estimations of case-fatality ratios for measles, to develop a prediction model to estimate case-fatality ratios across heterogeneous groupings, and to project future case-fatality ratios for measles up to 2030.

**Methods:**

We did a review of the literature to identify studies examining measles cases and deaths in low-income and middle-income countries in all age groups from 1980 to 2016. We extracted data on case-fatality ratios for measles overall and by age, where possible. We developed and examined several types of generalised linear models and determined the best-fit model according to the Akaike information criterion. We then selected a best-fit model to estimate measles case-fatality ratios from 1990 to 2015 and projected future case-fatality ratios for measles up to 2030.

**Findings:**

We selected 124 peer-reviewed journal articles published between Jan 1, 1980, and Dec 31, 2016, for inclusion in the final review—85 community-based studies and 39 hospital-based studies. We selected a log-linear prediction model, resulting in a mean case-fatality ratio of 2·2% (95% CI 0·7–4·5) in 1990–2015. In community-based settings, the mean case-fatality ratio was 1·5% (0·5–3·1) compared with 2·9% (0·9–6·0) in hospital-based settings. The mean projected case-fatality ratio in 2016–2030 was 1·3% (0·4–3·7).

**Interpretation:**

Case-fatality ratios for measles have seen substantial declines since the 1990s. Our study provides an updated estimation of case-fatality ratios that could help to refine assessment of the effect on mortality of measles control and elimination programmes.

**Funding:**

Bill & Melinda Gates Foundation.

## Introduction

In 2000, measles was a leading cause of vaccine-preventable child mortality, with an estimated global mortality burden of 535 000 deaths.^1^ Measles resulted in approximately 110 000 deaths annually, according to 2017 estimates.^2^ However, despite observed decreases in mortality in under-5s,^3^ we have not seen any noticeable changes in case-fatality ratio estimates for measles, which are used in the underlying assumptions for measles mortality models. These estimates might be expected to change as the characteristics of measles-susceptible populations evolve with immunisation coverage, as the age of infection increases, and as nutritional status improves.^4^, ^5^ For example, greater vaccination coverage has been known to increase the age of infection; this can subsequently lower mortality, which previous analyses have addressed with age-specific case-fatality ratios for measles.^5^, ^6^ Although several analyses have explored time trends in measles mortality estimates,^1^, ^2^, ^7^, ^8^, ^9^ many measles modelling efforts rely on a review^6^ of case-fatality ratios in low-income and middle-income countries (LMICs), published in 2009. The work reviewed age-specific case-fatality ratios for measles in community-based studies published between 1980 and 2008, but did not include hospital-based studies or analyse time trends.

Comprehensively reviewing and updating our knowledge of case-fatality ratios for measles will better account for the past health improvements and economic benefits driven by measles vaccination and for the potential improvements in the future.^10^, ^11^, ^12^ As a case in point, an analysis^11^ published in 2016 has estimated that immunisations done during 2011–20 would return an economic benefit approximately 16 times greater than cost and, within these estimates, preventing measles would return an economic benefit approximately 58 times greater than the cost. The assessment of the measles mortality burden in such estimations,^3^, ^7^ as well as the contribution of measles vaccination to mortality reductions, remains highly dependent on the estimates used for case-fatality ratios for measles, which can vary according to geography, health systems infrastructure, prevalence of underlying risk factors, and measles endemicity. With imprecise case-fatality ratios, there is continued uncertainty about the burden of measles mortality and the effect of measles vaccination, including its cost-effectiveness and return on investment.^13^

**Research in context**

**Evidence before this study**

We systematically reviewed the literature in PubMed to identify both community-based and hospital-based studies examining measles cases and deaths, published between Jan 1, 1980, and Dec 31, 2016. We identified 124 studies from which we obtained case-fatality ratios for measles in low-income and middle-income countries (LMICs) in all age groups. These studies used different study designs across different types of settings and were not always nationally representative. Few studies included information about laboratory confirmation of measles and there were varying definitions of the timeline following rash onset for a death to be considered attributable to measles. Many measles modelling efforts rely on a previous review of case-fatality ratios for measles in LMICs published in 2009, which reviewed age-specific case-fatality ratios in community-based studies published between 1980 and 2008. In our study, we build upon this previous review by re-examining literature published in 1980–2008, extending its scope to add articles published in 2008–16, and including hospital-based studies in our analysis.

**Added value of this study**

From the data gathered in our literature review, we developed a prediction model to estimate case-fatality ratios for measles across heterogeneous groupings (by country, income level, year, and age). We found that estimated case-fatality ratios for 1990–2015 would be about 2%, and projected case-fatality ratios would decline to about 1% by 2030. Overall, the estimated case-fatality ratios were highest in hospital settings and in children younger than 5 years, consistent with other analyses that reported a larger burden of measles mortality in this age group.

**Implications of all the available evidence**

Our study and model can help to improve predictions of the effect and cost-effectiveness of measles control and elimination programmes and can elucidate some of the uncertainty surrounding this effect.

In this Article, we build upon the previous review^6^ by re-examining the literature published in 1980–2008, extending the scope of the search to add articles published in 2008–16, and including hospital-based studies in our analysis. We first did a literature review of case-fatality ratios for measles in both community-based and hospital-based cases in LMICs from empirical data in the published literature from 1980–2016. We subsequently developed a prediction model to estimate case-fatality ratios across heterogeneous groupings (by country, income level, year, and age) from 1990–2015. Finally, we projected future case-fatality ratios for measles up to 2030.

## Methods

### Search strategy and selection criteria

We identified studies to supplement the previous review by Wolfson and colleagues^6^ by searching PubMed for community-based and hospital-based studies with primary data on measles cases and deaths from Jan 1, 1980, to Dec 31, 2016, in LMICs, as classified by 2017 World Bank income level. Primary data were defined as directly collected data from outbreak investigations, cohort studies, analyses of routine surveillance, cross-sectional studies, and hospital-based studies. The search strategy used a combination of controlled vocabulary (MeSH) terms and free text terms. For community-based studies, we used the following key search terms: (measles[MeSH Terms] OR measles) AND (mortality[MeSH Terms] OR mortality OR “case fatality rate” OR “case fatality ratio”). For hospital-based studies, the key search terms were the following: (measles[MeSH Terms] OR measles) AND (mortality[MeSH Terms] OR mortality OR “case fatality rate” OR “case fatality ratio”) AND (hospitals[MeSH Terms] OR hospital). We excluded articles that were not human studies, were outside the specified data range, contained outcomes from a study already reported in another article, did not include primary data, contained an abstract in a language other than English, or were otherwise considered irrelevant because of the study design, setting, or absence of information on measles cases and deaths. We also excluded articles that referred to measles outbreaks in camps of refugees or internally displaced people, per the methods described in Wolfson and colleagues.^6^ All studies identified in Wolfson and colleagues^6^ were included a priori and, therefore, removed as duplicates in the literature review. The full search strategy is described in the appendix.

### Data extraction and preliminary analysis

We extracted data on case-fatality ratios for measles overall and by age, where possible. The data extracted included study year, first-level administrative region (eg, state) in countries with populations greater than 50 million people, age group (if listed), setting (urban or rural, if available), number of cases, number of deaths, laboratory confirmation of measles (yes or no), and timeframe following the rash onset for death to be considered attributable to measles, if applicable. A summary table of laboratory confirmation and timeframe following rash onset is provided in the appendix. The case-fatality ratios included all suspected measles cases as defined in each included article. A measles death was defined according to the article, if a timeline was not given, or as any death within 30 days of rash onset, if such data were extractable. Therefore, any potential effect of measles on longer-term mortality, including the effect of subacute sclerosing panencephalitis, was beyond the scope of this review.^9^, ^14^

We extracted data from community-based and hospital-based cases separately because we expected the hospital-based case-fatality ratios to be higher, given that children are often hospitalised with measles because of increased severity and complications.^6^ We also extracted data by age, where possible, because the case-fatality ratios in children younger than 5 years were expected to be higher than those in individuals aged 5 years or older.^7^, ^15^

### Prediction model

First, we aggregated the following preliminary list of country-level covariates^1^, ^6^, ^15^ that were often associated with case-fatality ratios for measles in the literature: previous history of vaccination,^6^, ^15^ estimated measles incidence, approximate attack rate for measles (estimated measles incidence divided by annual birth cohort),^16^ and prevalence of HIV.^16^ Additionally, the following covariates were hypothesised to be indirectly associated with case-fatality ratios for measles: gross national income per capita,^16^ under-5 mortality,^16^ total fertility rate,^16^ percentage of population living in urban areas,^16^ population density (per km^2^ of land area),^16^ and educational attainment.^17^ Because we were unable to obtain individual history of vaccination from most studies, we used estimated coverage of the routine first dose of measles-containing vaccine (MCV1) as a proxy.^18^

We estimated the annual measles incidence for all countries by use of previously described methods^1^, ^19^ applied to reported measles cases from 1980 to 2014.^20^ Briefly, the input data used were reported annual cases of measles (as collected with the WHO joint reporting form), national estimates of coverage with the first and second dose of MCV, coverage of national and subnational supplementary immunisation activities, and annual UN estimates of birth rates and population size from 1980 to 2014. The incidence of unreported cases was estimated by use of an extended Kalman filter.^1^, ^19^ The Kalman filter, and its non-linear extension called extended Kalman filter, is a general method for fitting partly observed time-series processes (eg, underreported disease incidence through time).^19^ This model was fitted independently to each country, resulting in country-specific estimates of the reporting rate and estimates of the unreported cases.

We obtained each covariate for the year of each study, with the midpoint year used for studies done across multiple years. We also included in the model the study year (as a continuous variable) and dummy indicators for community-based studies and case-fatality ratios in children younger than 5 years. The extracted case-fatality ratios for measles from the studies were age specific, where available. Therefore, the under-5 dummy indicator was used only for case-fatality ratios that were specific to children younger than 5 years. Likewise, this dummy indicator was not used for case-fatality ratios in individuals aged 5 years or older. For studies that did not provide age-specific case-fatality ratios, the dummy indicator was not applied. For studies that reported more than one case-fatality ratio—including one or more age-specific ratios, ratios across one or more timeframes, and so on—the analysed data incorporated each individual case-fatality ratio observation.

Because several studies did not disaggregate by age, but were likely to represent a mixture of ages older or younger than 5 years, we ran the model without these studies and then ran two scenario analyses: one assuming that these studies' case-fatality ratios were attributable to children younger than 5 years, and another assuming that they were attributable to individuals aged 5 years or older.

Afterwards, we examined several types of generalised linear models (ie, log-linear, Poisson, quasi-Poisson, and negative binomial) to fit the dataset of case-fatality ratios for measles in all LMICs across 1990–2015. Our model predicts case-fatality ratios from 1990 onwards because measles control efforts in this period are more relevant to current efforts than those during the 1980s, which were markedly different, with MCV1 coverage ramping up, no supplementary immunisation activities or routine second-dose coverage, and large measles outbreaks. We used an offset term (measles incidence) to account for over-dispersion in the Poisson model. Because of overlap and relevance of the preliminary list of covariates, we removed covariates with more than 10% of data missing and subsequently examined model fit with the remaining covariates.

Although not all doses of MCV are delivered through routine services, comprehensive data were not available to capture additional coverage from supplementary immunisation activities. Similarly, patient-level factors that are associated with measles mortality risk, such as vaccination, nutritional, and treatment status, were not comprehensively available for individual cases in most included studies. Therefore, these patient-level factors were not included in the prediction model.

Given the importance of a prediction model that produces results with limited data, we determined the best-fit model according to the Akaike information criterion. Because selected studies were of varying quality and size, the analysis weighted the model estimation by the number of measles cases from each study. The best-fit model was validated by a cross-validation method of training the model on 80% of the dataset and testing the predictive capacity of the model on the remaining 20% (appendix).

### Projection of future case-fatality ratios

After using the prediction model to estimate case-fatality ratios for measles in 1990–2015, we used a projection model for future case-fatality ratio estimations with the covariates for which projected estimates were available up to 2030. These included covariates were the following: year, an indicator for community-based studies, an indicator for studies in children younger than 5 years, MCV1 coverage,^18^ under-5 mortality,^21^ total fertility rate,^21^ percentage of population in urban areas,^21^ and population density.^21^ Population density was available with annual projections, whereas under-5 mortality, total fertility rate, and urban percentage were available only by 5-year increments.^21^ We projected future MCV1 coverage on the basis of 2015 WHO-UNICEF estimates of coverage,^18^ assuming a 1% coverage increase per year, in line with previous analyses of Gavi, the Vaccine Alliance.^22^ Future MCV1 coverage was capped at 95%, unless a country had a higher projected coverage as of 2015, in which case the coverage was capped at the maximum coverage reached. We also included a sensitivity analysis with MCV1 coverage capped at 90% and holding WHO's estimated MCV1 coverage for 2015 constant across the 2016–30 projection period.

To address model uncertainty in our estimations, we explored the distribution of predicted and projected case-fatality ratios when drawing simulated regression coefficients from a multivariate normal distribution (n=1000), with use of the model's estimated coefficients and variance–covariance matrix. Equations of the fitted, prediction, and projection models, with accompanying definitions and sources, are presented in the appendix. We used R statistical software, version 3.5.1, for all analyses.

### Role of the funding source

The funder of the study had no role in study design, data collection, data analysis, data interpretation, or writing of the report. The corresponding author had full access to all the data in the study and had final responsibility for the decision to submit for publication.

## Results

**Figure 1:**
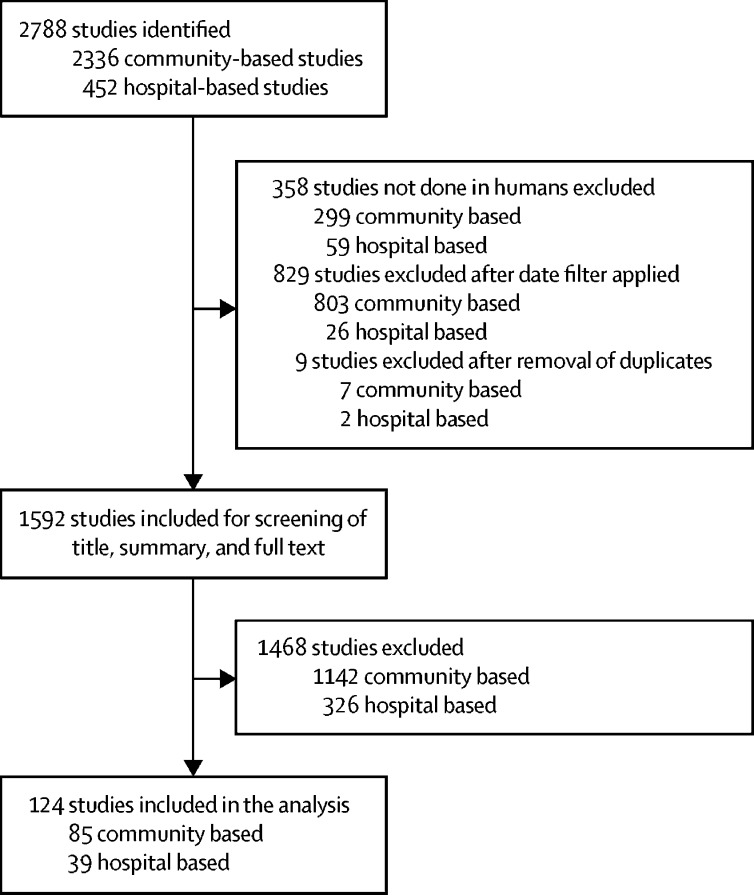
Review of the literature published in 1980–2016 of case-fatality ratios for measles in low-income and middle-income countries

We identified 2788 articles in our initial literature review (figure 1). After filtering for human studies across the relevant timeframe (studies published between Jan 1, 1980, and Dec 31, 2016) and excluding duplicates, 1592 of these articles were considered potentially relevant. The titles and abstracts of these remaining articles were further screened for inclusion in the review. Full texts were reviewed only if no clear exclusion criteria appeared in the abstract. Articles that discussed clinical trials, measles outbreaks in refugee camps or camps of internally displaced people, measles in developed countries (defined as high-income countries, with a gross national income per capita of US$12 476 or higher^23^), or otherwise did not include measles deaths and cases in LMICs were excluded. A total of 124 articles were selected for inclusion in the final review—85 community-based studies and 39 hospital-based studies. A full list of the studies included is provided in the appendix. Three review articles were selected for community-based studies,^24^, ^25^, ^26^ but original sources were obtained where available (19 additional original sources obtained and four not available). Case-fatality ratios in the original sources were prioritised over the data in the published reviews, where applicable. One study included both community-based and hospital-based measles cases and was included in both datasets.^27^ The community-based studies contained 158 observations across 35 countries (including 68 observations in children younger than 5 years, 41 in individuals aged 5 years or older, and 49 that were not disaggregated by age), whereas hospital-based studies contained 68 observations across 23 countries (24 observations in under-5s, 12 in individuals aged 5 years or older, and 32 that were not disaggregated by age).

**Figure 2:**
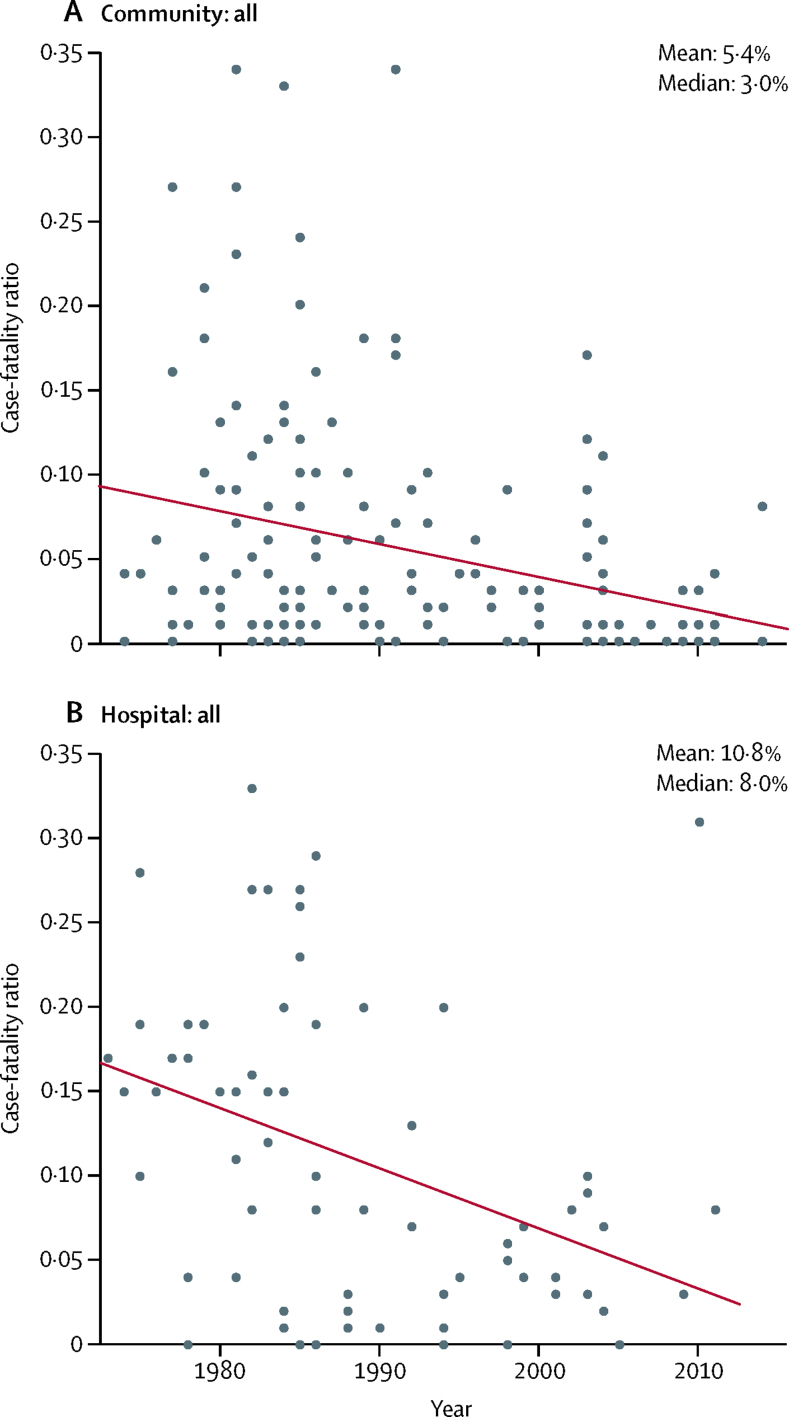
Case-fatality ratio for measles, by year of study, extracted from studies published in 1980–2016 Line indicates the trend line of the case-fatality ratio for measles by year, according to a simple linear regression of case-fatality ratio with time (year). 158 observations from community-based studies; 68 observations from hospital-based studies.

Of the 226 case-fatality ratio observations for measles, 65 were done in low-income country settings and 161 were done in middle-income country settings, as classified by 2017 World Bank income level. The unweighted median case-fatality ratio in community-based studies was 3·0% (mean 5·4%). The median case-fatality ratio in hospital-based studies was 8·0% (mean 10·8%). A histogram of the case-fatality ratios reported for all age-groups in all studies is included in the appendix. The number of measles cases in all community-based and hospital-based studies analysed was 523 885. The number of measles cases by study varied considerably, with a median of 349 cases in community-based studies (range 8–124 865) and a median of 357 in hospital-based studies (range 29–7447). Figure 2 shows the range of measles case-fatality ratio observations by year of study (results by income level are included in the appendix).

The time period of the studies tended to be more recent in community-based studies than in hospital-based studies, but, overall, tended towards the beginning of the search period of our study, with a midpoint year mean of 1991 (median 1989) among community-based studies and of 1989 (median 1986) among hospital-based studies. The majority of community-based studies were done in rural areas (59%), whereas nearly half of hospital-based studies were done in urban areas (48%), with only three studies done in rural areas and the remaining studies done in both urban and rural areas. For nine studies done at the national level, the urban or rural indicator was assigned according to the percentage of population in urban areas, resulting in two studies classified as urban and seven studies classified as rural.^16^ A majority of community-based studies were done during measles outbreaks (67%), whereas a minority of hospital-based studies took place during outbreaks (19%). The largest community-based studies took place in China, Democratic Republic of the Congo, and Malawi (62% of all measles cases included in the community-based analysis)—all of which occurred in outbreak settings.

According to model selection and goodness of fit tests, we selected a log-linear model (table 1). The Poisson models had non-significant p values on tests for over-dispersion, indicating that a quasi-Poisson model was not necessary. For the final model, we also eliminated covariates with more than 10% of data missing across the 136 LMICs, but non-significant covariates were preserved as relevant to the prediction of case-fatality ratio. The model used to predict case-fatality ratios for measles during 1990–2015 relied on study year, MCV1 coverage, an indicator for community-based studies, an indicator for studies in children younger than 5 years, measles attack rate, under-5 mortality, population density, total fertility rate, and percentage of population in urban areas. The regression results are presented in the appendix. The log-linear model selected posits that these covariates predict case-fatality ratios for measles by acting as proxies for how case-fatality ratios are likely to differ across time and geography. The model achieves this by assuming that case-fatality ratio estimates scale by study year, measles vaccine coverage, and country-level differences based on the distribution and health of the population (eg, under-5 mortality, population density, and so on). The final model that we used to predict case-fatality ratios had *R*^2^=0·74. A direct comparison of observed versus predicted case-fatality ratio values is shown in the appendix.

**Table 1:** Model goodness of fit for the 1990–2015 generalised linear models predicting measles case-fatality ratios in low-income and middle-income countries

	**Akaike information criterion**
**Models with all covariates**	
Log linear	10
Poisson	3774
Negative binomial	1011
Models with covariate subset*	
Log linear	−321
Poisson	4548
Negative binomial	1013
Best-fit model selected	−337

Best-fit model selected is log linear. Equations of models are listed in the appendix.

* The subset included all covariates that had less than 10% of data missing (ie, covariates with 10% or more of data missing were excluded).

**Figure 3:**
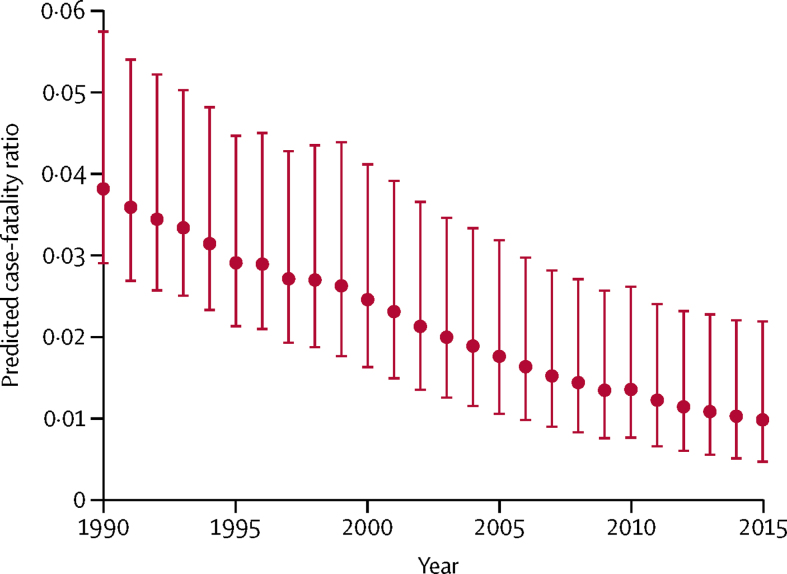
Mean predicted case-fatality ratios for measles from community-based and hospital-based studies, 1990–2015 Predicted case-fatality ratios (dots) are averaged arithmetically across all 136 low-income and middle-income countries for all ages in each year to provide a mean predicted case-fatality ratio in that year, with bounds according to the 95% CI. Community-based and hospital-based studies were weighted by the number of measles cases from each included study.

**Figure 4:**
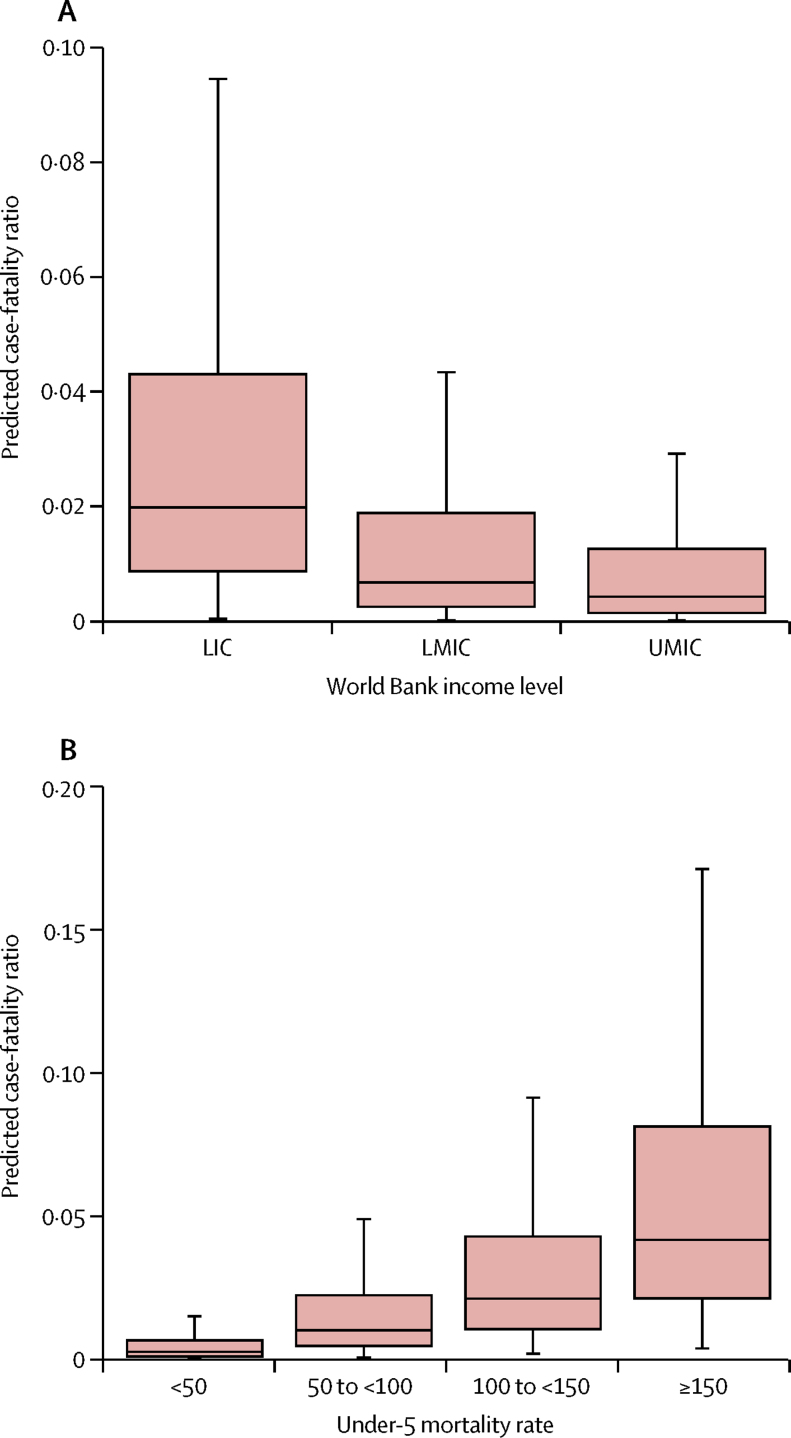
Boxplots of predicted case-fatality ratios for measles from 1990–2015 by World Bank income level (A) and under-5 mortality (B) for 136 low-income and middle-income countries and all ages Under-5 mortality defined as the estimated number of deaths per 1000 livebirths. LIC=low-income country. LMIC=lower-middle-income country. UMIC=upper-middle-income country.

The mean predicted case-fatality ratio for 1990–2015 was 2·2% (95% CI 0·7–4·5). In community-based case-fatality ratios, the mean predicted ratio was 1·5% (0·5–3·1), compared with 2·9% (0·9–6·0) in hospital-based ratios. In children younger than 5 years, the mean predicted ratio was 3·3% (1·1–6·4) across this period, compared with a mean predicted ratio of 0·9% (0·2–3·4) in individuals aged 5 years or older. Table 2 shows the estimated case-fatality ratios for measles categorised by under-5 mortality and by setting. Figure 3 shows the mean predicted case-fatality ratio in all countries for 1990–2015 (the predicted ratio unweighted by the number of measles cases from each literature review study and the population-averaged predicted ratio are included in the appendix). Although the predicted case-fatality ratios included outliers, only 0·3% of the estimated values were greater than the maximum case-fatality ratio found in the literature reviewed. When comparing children by age group, predicted case-fatality ratios were highest in under-5s, but our analysis resulted in lower mean predicted case-fatality ratios in under-5s in 1990–2015 compared with those in the original data in 1980–2016 (appendix). The estimated case-fatality ratios by World Bank income level were, as expected, highest in low-income countries and decreased as gross national income per capita increased (figure 4). Likewise, the predicted case-fatality ratios decreased as under-5 mortality decreased (figure 4).

**Table 2:** Measles case-fatality ratio median predictions and minimum–maximum ranges by setting and by under-5 mortality and income level, for the 1990–2015 period and for 2015 alone

		**Overall CFR**	**Community-based CFR**	**Hospital-based CFR**	**Under-5 CFR**	**Over-5 CFR**
**1990–2015**						
Under-5 mortality*						
	<50	1·1% (0·0–8·2)	0·7% (0·0–4·6)	1·4% (0·0–11·9)	1·6% (0·0–8·9)	0·4% (0·0–9·1)
	50 to <100	4·0% (0·0–14·4)	2·7% (0·0–10·3)	5·2% (0·0–19·8)	6·1% (0·0–17·7)	1·6% (0·0–16·5)
	100 to <150	6·6% (0·0–20·9)	4·4% (0·0–13·8)	8·6% (0·0–30·0)	10·1% (0·0–30·7)	2·6% (0·0–22·7)
	≥150	7·6% (0·0–28·4)	5·2% (0·0–16·7)	10·0% (0·0–40·9)	11·8% (0·0–41·7)	3·3% (0·0–26·0)
Income level†						
	LIC	3·6% (0·0–14·0)	2·4% (0·0–9·8)	4·7% (0·0–19·0)	5·4% (0·0–17·5)	1·5% (0·0–16·8)
	LMIC	2·0% (0·0–9·4)	1·4% (0·0–5·8)	2·7% (0·0–13·5)	3·1% (0·0–10·8)	0·8% (0·0–10·4)
	UMIC	1·5% (0·0–10·0)	1·0% (0·0–5·6)	1·9% (0·0–14·4)	2·2% (0·0–11·7)	0·6% (0·0–11·1)
**2015**						
Under-5 mortality*						
	<50	0·4% (0·1–3·5)	0·3% (0·1–2·4)	0·5% (0·1–4·6)	0·6% (0·2–5·8)	0·2% (0·0–3·2)
	50 to <100	1·8% (0·6–6·9)	1·2% (0·4–4·7)	2·4% (0·8–9·1)	2·8% (0·9–11·4)	0·8% (0·1–6·0)
	100 to <150	3·5% (1·2–12·4)	2·4% (0·8–7·2)	4·6% (1·3–18·0)	5·3% (1·7–18·5)	1·5% (0·2–11·7)
	≥150	6·2% (2·0–20·3)	4·2% (1·4–12·3)	8·2% (2·7–29·3)	9·6% (3·2–30·1)	2·7% (0·3–18·9)
Income level†						
	LIC	1·6% (0·5–5·0)	1·1% (0·4–3·4)	2·1% (0·7–7·1)	2·4% (0·8–8·3)	0·7% (0·1–3·4)
	LMIC	0·9% (0·3–4·4)	0·6% (0·2–3·0)	1·2% (0·4–5·8)	1·4% (0·5–7·3)	0·4% (0·1–3·8)
	UMIC	0·7% (0·2–5·0)	0·5% (0·1–3·4)	0·9% (0·2–6·6)	1·1% (0·3–8·3)	0·3% (0·1–4·3)

The 2015 case-fatality ratio (CFR) ranges represent the minimum and maximum CFR by country from the specified grouping. The 1990–2015 CFR ranges represent the minimum minus SD (or 0 if the CFR becomes negative) and the maximum plus SD from the specified grouping, to reflect the uncertainty in the regression model. LIC=low-income country. LMIC=lower-middle-income country. UMIC=upper-middle-income country.

* Under-5 mortality is defined as the estimated number of deaths of children younger than 5 years per 1000 livebirths; countries are classified by 2015 under-5 mortality.

† As classified by 2017 World Bank income level.

In the first scenario analysis, we assumed that the 81 case-fatality ratios from studies that were not disaggregated by age were attributable to children younger than 5 years: this resulted in a mean predicted ratio for 1990–2015 of 1·8% (95% CI 0·6–3·8). In the second scenario analysis, we assumed that the 81 case-fatality ratios from studies that were not disaggregated by age were attributable to children aged 5 years or older: this resulted in a mean ratio of 2·5% (1·0–4·4).

Because not all prediction model covariates had projected estimates available in 2016–30 (ie, attack rate), we relied on a projection model with adjusted covariates. A comparison between the goodness of fit statistics of the prediction model and the projection model is presented in the appendix. With only one less parameter (attack rate) than the prediction model, the projection model showed better fit (−323) compared with most examined models, but worse fit compared with the prediction model (−337), according to the Akaike information criterion.

The mean projected case-fatality ratio for 2016–30 was 1·3% (95% CI 0·4–3·7). In community-based case-fatality ratios, the mean projected ratio was 0·8% (0·3–2·2), compared with 1·8% (0·6–5·4) in hospital-based case-fatality ratios. In children younger than 5 years, the mean projected ratio was 1·9% (0·7–5·2), compared with a mean projected ratio of 0·6% (0·1–2·5) in individuals aged 5 years or older.

**Figure 5:**
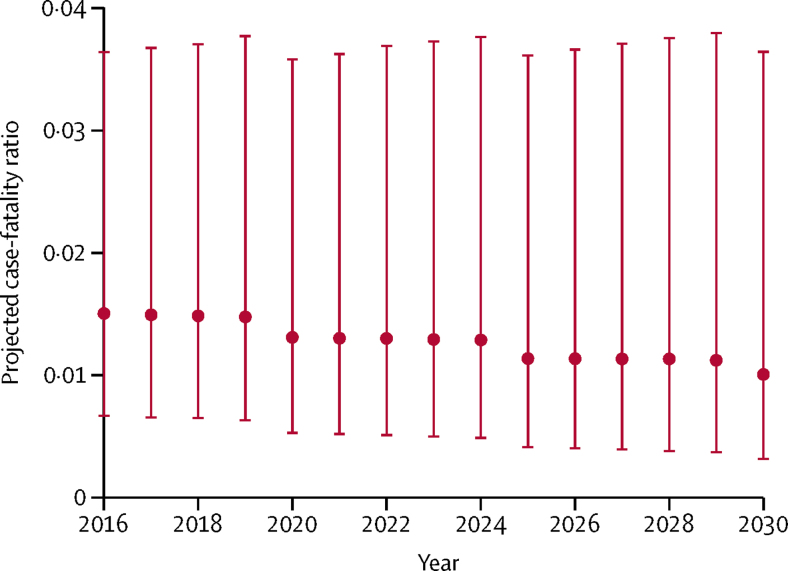
Mean projected case-fatality ratios for measles from community-based and hospital-based studies, 2016–2030 Projected case-fatality ratios (dots) are averaged arithmetically across all 136 low-income and middle-income countries for all ages in each year to provide a mean projected case-fatality ratio in that year, with bounds according to the 95% CI. Community-based and hospital-based studies were weighted by the number of measles cases from each included study.

When examining projected case-fatality ratios for 2016–30 in the sensitivity analysis capping MCV1 coverage at 90%, rather than 95%, we found that the mean projected case-fatality ratio overall remained at 1·3% (95% CI 0·4–3·6), whereas the mean projected ratio in children younger than 5 years increased slightly to 2·0% (0·7–5·3) compared with a projection capping MCV1 coverage at 95%. In the sensitivity analysis holding MCV1 at constant 2015 coverage levels, we also found that the mean projected case-fatality ratio overall remained the same compared with capping coverage at 95%. Figure 5 shows the mean projected case-fatality ratio in 2016–30 (projected case-fatality ratios by under-5 mortality are included in the appendix).

## Discussion

In our study, we did a literature review of case-fatality ratios for both community-based and hospital-based measles cases from the published literature in LMICs to develop a prediction model to estimate case-fatality ratios for measles. We found that estimated case-fatality ratios for 1990–2015 would be about 2%, and projected case-fatality ratios would decline to about 1% by 2030. Overall, the estimated case-fatality ratios were highest in hospital settings and in children younger than 5 years, consistent with other analyses that reported a larger burden of measles mortality in this age group.^15^

Our analysis attempted to address the uncertainty surrounding case-fatality ratios for measles and to better estimate the burden of measles mortality. Our case-fatality ratio estimates are a stepping stone towards improving mortality estimations, particularly for the WHO burden of disease assessments, including their tracking progress toward measles control, elimination, and mortality reduction. In concertation with academics and technical experts, our work could enable us to provide case-fatality ratio estimates as inputs into the modelling projections of WHO.^1^, ^20^

Our study found lower mean case-fatality ratios in community-based studies compared with those found in the previous descriptive analysis done by Wolfson and colleagues,^6^ both in the articles we reviewed and in our estimations. Our study reviewed articles published from 1980 to 2016, whereas Wolfson and colleagues^6^ relied on articles published from 1980 to 2008. Our review identified six additional articles between 1980 and 2008 that were not captured by Wolfson and colleagues,^6^ as well as 26 articles between 2008 and 2016. Additionally, our analysis examined both community-based and hospital-based studies, whereas the previous work included only community-based studies (appendix). Our analysis also went beyond the previous descriptive analysis by developing a model to provide estimations of case-fatality ratios.

Previous models of measles burden and vaccine impact, including dynamic transmission models, have attempted to estimate the burden of measles mortality globally. Although some models directly relied on verbal autopsy data,^28^ many models estimated measles burden by applying age-specific and country-specific case-fatality ratios to the values estimated in the associated model.^1^, ^2^, ^29^ In this respect, our analysis addressed these underlying limitations by focusing on the case-fatality ratio inputs and providing selected disaggregations of case-fatality ratio estimates (including variation by time and setting).

Nevertheless, our analysis has several limitations. First, we assumed that case-fatality ratio data from each published study were nationally representative. We also assumed that all studies that included measles cases and deaths in individuals aged 5 years or older could be grouped and analysed together, although the age ranges were not consistently or transparently defined in each study. However, because of the small number of studies, we chose to include more data in our analysis rather than to reduce our dataset further. We also included some analyses of routine surveillance data (12% of all selected study observations) that directly reported on primary data collection. However, because routine surveillance might not capture all outcomes associated with cases, including deaths, our case-fatality ratio estimates for measles might be biased. Moreover, these studies used different study designs across different types of settings, and the exclusion of four studies in refugees and displaced populations might have biased the estimates towards lower case-fatality ratios, because these are typically high-risk populations. The inclusion of uncertainty and scenario analyses aimed to address these limitations by estimating uncertainty intervals. However, additional uncertainty due to limitations and quality in the original data might have remained. Additionally, although 43 countries were represented in the original dataset, five countries (Ghana, Guinea-Bissau, India, Nigeria, and Senegal) represented 46% of all 226 observations. When considering the number of cases used in weighting the final model, three countries (China, Democratic Republic of the Congo, and Malawi) represented 62% of all cases. However, most of these cases have been included in studies since 2000. Additionally, when running the model without these countries' observations, the mean estimated case-fatality ratio in 1990–2015 increased from 2·2% to 2·5%. Overall, the majority of the observations included were also based on data from the 1970s and 1980s, with approximately 55% of the observations having a midpoint study year earlier than 1990. Moreover, the studies since 2000, which represent 23% of the observations in this study, did not show as clear a declining trend as seen in earlier years. However, case-fatality ratios higher than 10% during this period represent less than 3% of cases in our study, and when these outliers were removed, the declining trend was more pronounced.

A second limitation of our study was the existence of several factors that could not be addressed in our modelling approach because of data constraints, including a previous history of vaccination at the individual level, a subgroup analysis for individuals with HIV infection, the effect of malnutrition, and the effect of improved measles case management. However, we used estimated coverage of MCV1 as a proxy for individual vaccination status, which might not capture the heterogeneity at the individual level. Because of the number of countries with high prevalence of HIV included among LMICs, the increased mortality from measles among individuals with HIV infection is a relevant, but unaddressed factor in our analysis.^30^ Malnutrition also contributes to measles mortality, and improvements in vitamin A deficiencies and nutrition over time might also support the declining case-fatality ratio trend for measles.^31^ Additionally, measles case management has improved since 1980, and is probably a further contributing factor to subsequent improvements in measles mortality.^32^, ^33^, ^34^

A third limitation was the varying quality in the articles included. Few studies included information about laboratory confirmation of measles, and there were varying definitions of the timeline following rash onset for a death to be considered attributable to measles (appendix). Approximately 50% of the community-based studies included in the analysis defined the timeline as 1 month following rash onset (ie, 1 month, 4 weeks, 28 days, 30 days, or 31 days). However, hospital-based studies typically had a follow-up on a much shorter timeline and, therefore, might have missed deaths according to this definition. For both community-based and hospital-based studies, the period of measurement of death, as well as the completeness of ascertainment of deaths, was not standardised and often not known. We assumed that the suspected measles cases and deaths as defined in each article were sufficient to estimate the case-fatality ratio for measles, which might have introduced bias if either the number of suspected cases was greater than the true number of measles cases or if the deaths attributable to measles were greater than the timeline by which data were defined and collected. Although our analysis weighted case-fatality ratios in studies by the number of cases, we also could have used a grading scheme that incorporated some sense of the quality of each study. To address the specific issues related to quality in the context of each study, we would need to develop some kind of weighting based on criteria such as laboratory confirmation, loss to follow-up, and time following rash onset for death to be attributable to measles not defined. However, given the scarcity of detailed information in each study, this approach was impractical and we chose to rely on the number of measles cases alone.

A fourth limitation was that the models presented here do not represent a causal relationship between the covariates of interest and the outcome. Additional factors that are important considerations for mortality risk—such as vaccination, nutritional, and treatment status of the individual cases—were not included because of absence of patient-level data in the study reports. Our study also needs comprehensive information on supplementary immunisation activities. Because of this need, measles immunisation coverage in this study reflected only routine immunisation coverage and not the supplemental coverage of the country at the time of data collection for each included article. We also optimistically projected MCV1 coverage by capping it at 95% (although 50 LMICs had achieved 95% or higher MCV1 coverage as of 2015, according to WHO estimates^18^), so as to not arbitrarily limit coverage estimates by 2030 and to reflect the higher expected coverage due to supplementary immunisation activities. Additionally, for both prediction and projection models, we assumed that the relationship between the analysed covariates and case-fatality ratios for measles have the same functional form over time. Although we did incorporate more than one observation per study for some studies that included age-specific case-fatality ratios in both under-5s and individuals aged 5 years or older, this was done only for a minority of the studies (approximately 30%). Finally, because the attack rate covariate was excluded from the projection model and the relationship between attack rate and case-fatality ratio for measles is positive, we might expect that the potential information loss in the projection model could result in underestimation of case-fatality ratios for measles.

Nonetheless, our analysis enabled us to draw several conclusions. First, as expected, hospital-based measles cases, which are likely to be more severe, were associated with higher case-fatality ratios compared with those of community-based measles cases. Second, the highest case-fatality ratios were found in under-5s, the most susceptible group and with the highest measles mortality. Third, as under-5 mortality—a population-level predictor of case-fatality ratio—decreases, case-fatality ratio logically decreases.

International organisations such as WHO, UNICEF, the Measles & Rubella Initiative, and Gavi provide support and subsidies for measles immunisation. However, despite this widespread support, many countries have still not achieved the WHO target of 95% reduction in measles mortality between 2000 and 2015.^35^ Our data serve to help improve predictions of the effect of measles control and elimination programmes and can serve to elucidate some of the uncertainty surrounding this effect.
