# Supplementary material for: Estimates of case-fatality ratios of measles in low-income and middle-income countries: a systematic review and modelling analysis
Source: Lancet Glob Health. 2019 Feb 21;7(4):e472–81. doi: 10.1016/S2214-109X(18)30537-0 (PMC6418190; doi:10.1016/S2214-109X(18)30537-0)
Supplement: Supplementary appendix [file mmc1.pdf]

THE LANCET
Global Health

Supplementary appendix

This appendix formed part of the original submission and has been peer reviewed. We post it as supplied by the authors.

Supplement to: Portnoy A, Jit M, Ferrari M, Hanson M, Brenzel L, Verguet S. Estimates of case-fatality ratios of measles in low-income and middle-income countries: a systematic review and modelling analysis. *Lancet Glob Health* 2019; published online Feb 20. http://dx.doi.org/10.1016/S2214-109X(18)30537-0.

Supplementary appendix

Table of Contents

Appendix A. Literature review search strategy 2

Table B. Summary table of laboratory confirmation and definition of timeframe following rash onset for death to be considered attributable to measles, among community-based and hospital-based studies 3

Figure C. Comparisons of observed vs. predicted measles case fatality ratios (CFRs) 4

Appendix D. Equations of fitted, prediction, and projection models listed in Table 1 with accompanying definitions and sources 5

Appendix E. Community-based studies* included in the measles case fatality ratio (CFR) analysis 10

Appendix F. Hospital-based studies* included in the measles case fatality ratio (CFR) analysis 16

Figure G. Histogram of overall measles case fatality ratios (CFRs), extracted from the 1980-2016 review of the literature. 19

Figure H. Measles case fatality ratio (CFR) by country group income level and year of study, extracted from the 1980-2016 review of the literature. 20

Figure I. Mean predicted* measles case fatality ratios (CFRs) in each year when the CFR estimate from each study from the literature review is unweighted in the log-linear model estimation. 21

Figure J. Mean predicted* measles case fatality ratios (CFRs) in each year: population-averaged over 136 low- and middle-income countries, all ages. 22

Figure K. Boxplot comparing original* to predicted** measles case fatality ratios (CFRs) (below or above age 5) 23

Table L. Information loss between 2016–2030 projection and 1990–2015 prediction models 24

Figure M. Boxplot of projected measles case fatality ratios (CFRs) from 2016-2030 by under-5 mortality rate category for 136 low- and middle-income countries, all ages. 25

Table N. Comparison to the findings of Wolfson and colleagues' review of measles case fatality ratios 26

Appendix A. Literature review search strategy

Search strategy for community-based studies:

Initially include all 1980–2008 articles from Wolfson and colleagues[1]

Search terms for literature review supplementing Wolfson and colleagues:
(measles[MeSH Terms] OR measles) AND (mortality[MeSH Terms] OR mortality OR "case fatality rate" OR "case fatality ratio")

Exclusion criteria:

- Non-human studies
- Articles published outside data range (from 1980/01/01 to 2016/12/31)
- Included in Wolfson and colleagues
- Duplicate articles
- Did not include primary data (defined as directly collected data from outbreak investigations, cohort studies, analyses of routine surveillance, cross-sectional studies, and hospital-based studies)
- Non-English abstract
- Articles that referred to measles outbreaks in refugee camps or camps of internally displaced persons
- Irrelevant articles: clinical trials, global or regional surveillance, laboratory study, maternal mortality study, high-income countries, hospital study, no information on measles cases/deaths

Search strategy for hospital-based studies:

Search terms:
(measles[MeSH Terms] OR measles) AND (mortality[MeSH Terms] OR mortality OR "case fatality rate" OR "case fatality ratio") AND (hospitals[MeSH Terms] OR hospital)

Exclusion criteria:

- Non-human studies
- Articles published outside data range (from 1980/01/01 to 2016/12/31)
- Duplicate articles
- Did not include primary data (defined as directly collected data from outbreak investigations, cohort studies, analyses of routine surveillance, cross-sectional studies, and hospital-based studies)
- Non-English abstract
- Irrelevant articles: clinical trials, global or regional surveillance, laboratory study, maternal mortality study, high-income countries, community studies, no information on measles cases/deaths

Table B. Summary table of laboratory confirmation and definition of timeframe following rash onset for death to be considered attributable to measles, among community-based and hospital-based studies

	Laboratory confirmation	Definition of timeframe following rash onset for death to be considered attributable to measles included
Community-based articles	39 out of 158 observations	89 out of 158 observations
Hospital-based articles	13 out of 68 observations	12 out of 68 observations*

*Note: We assumed that, when not explicitly defined, hospital-based observations only included measles deaths in hospital.

Figure C. Comparisons of observed vs. predicted measles case fatality ratios (CFRs)

Figure C1. Cross validation for 20% of original measles CFR observations left out of prediction model

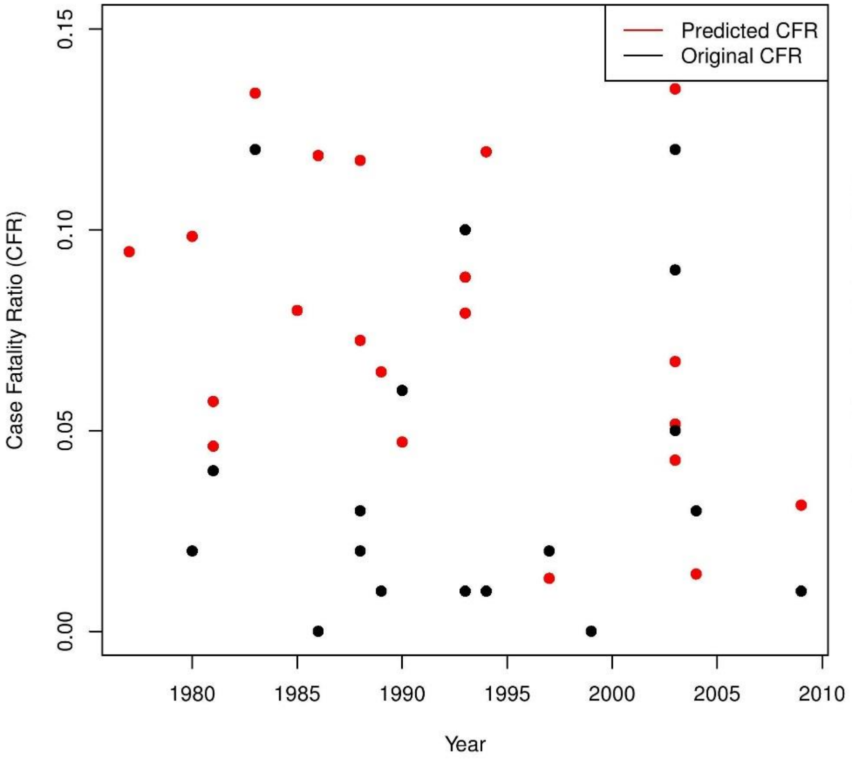

Figure C2. Observed vs. predicted measles CFRs for 1990–2015

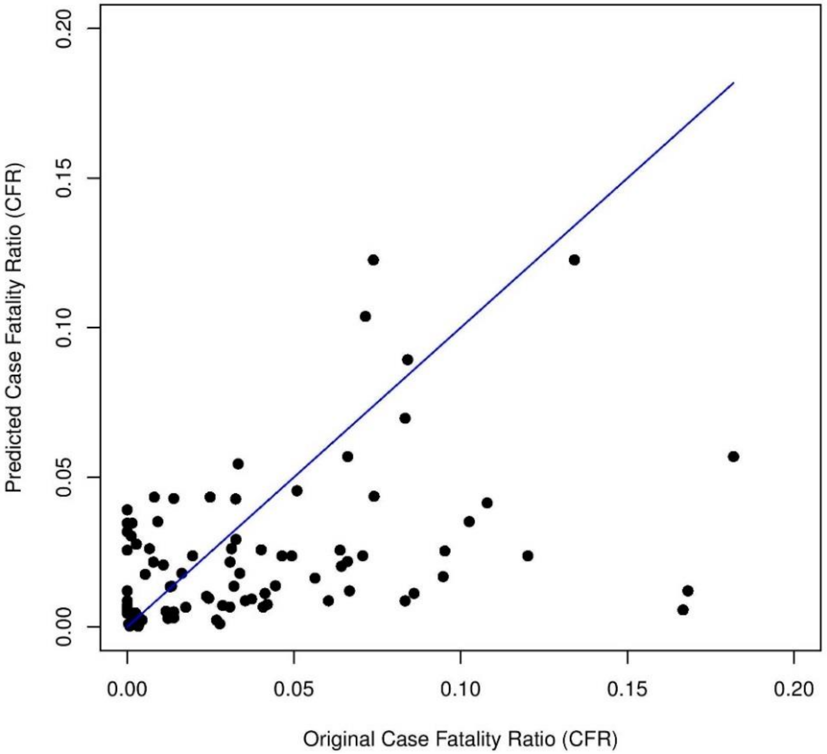

Note: Blue line indicates x=y. Original data includes only those CFRs from 1990 or later.

Appendix D. Equations of fitted, prediction, and projection models listed in Table 1 with accompanying definitions and sources

Fitted model equations including all covariates

<u>Log-linear</u>

$\ln(CFR) \sim Norm(\ln(\mu), \sigma^2)$,

where $\ln(\mu) =$

$$\beta_0 + \beta_1 * Year\ of\ study + \beta_2 * MCV1\ coverage + \beta_3 * Community\ indicator + \beta_4 * Under\ 5\ indicator + \beta_5 * Outbreak\ setting + \beta_6 * Attack\ rate + \beta_7 * \ln(GDP) + \beta_8 * \ln(Under\ 5\ mortality) + \beta_9 * \ln(Population\ density) + \beta_{10} * \ln(TFR) + \beta_{11} * Percentage\ urban + \beta_{12} * Region + \beta_{13} * Income\ level + \beta_{14} * Educational\ attainment + \beta_{15} * Urban\ setting + \beta_{16} * SIA + \beta_{17} * HIV$$

and $\sigma^2 =$ variance of $\ln(CFR)$

<u>Poisson</u>

$Deaths \sim Poisson(\lambda)$,

where $\ln(\lambda) =$

$$\beta_0 + \beta_1 * Year\ of\ study + \beta_2 * MCV1\ coverage + \beta_3 * Community\ indicator + \beta_4 * Under\ 5\ indicator + \beta_5 * Outbreak\ setting + \beta_6 * Attack\ rate + \beta_7 * \ln(GDP) + \beta_8 * \ln(Under\ 5\ mortality) + \beta_9 * \ln(Population\ density) + \beta_{10} * \ln(TFR) + \beta_{11} * Percentage\ urban + \beta_{12} * Region + \beta_{13} * Income\ level + \beta_{14} * Educational\ attainment + \beta_{15} * Urban\ setting + \beta_{16} * SIA + \beta_{17} * HIV$$

<u>Negative binomial</u>

$Deaths \sim NBin(r, p)$,

where mean $= \frac{pr}{1-p} =$

$$\beta_0 + \beta_1 * Year\ of\ study + \beta_2 * MCV1\ coverage + \beta_3 * Community\ indicator + \beta_4 * Under\ 5\ indicator + \beta_5 * Outbreak\ setting + \beta_6 * Attack\ rate + \beta_7 * \ln(GDP) + \beta_8 * \ln(Under\ 5\ mortality) + \beta_9 * \ln(Population\ density) + \beta_{10} * \ln(TFR) + \beta_{11} * Percentage\ urban + \beta_{12} * Region + \beta_{13} * Income\ level + \beta_{14} * Educational\ attainment + \beta_{15} * Urban\ setting + \beta_{16} * SIA + \beta_{17} * HIV$$

and variance $= \frac{pr}{(1-p)^2} =$ variance of the above

Fitted model equations including the covariates with less than 10% data missing

Log-linear

$\ln(CFR) \sim Norm(\ln(\mu), \sigma^2)$,

where $\ln(\mu) =$

$$\beta_0 + \beta_1 * Year\ of\ study +$$
$$\beta_2 * MCV1\ coverage + \beta_3 * Community\ indicator + \beta_4 * Under\ 5\ indicator + \beta_5 * Attack\ rate + \beta_6 * \ln(GDP) +$$
$$\beta_7 * \ln(Under\ 5\ mortality) + \beta_8 * \ln(Population\ density) + \beta_9 * \ln(TFR) + \beta_{10} * Percentage\ urban + \beta_{11} * Region + \beta_{12} * Income\ level$$

and $\sigma^2 =$ variance of $\ln(CFR)$

Poisson

$Deaths \sim Poisson(\lambda)$,

where $\ln(\lambda) =$

$$\beta_0 + \beta_1 * Year\ of\ study +$$
$$\beta_2 * MCV1\ coverage + \beta_3 * Community\ indicator + \beta_4 * Under\ 5\ indicator + \beta_5 * Attack\ rate + \beta_6 * \ln(GDP) +$$
$$\beta_7 * \ln(Under\ 5\ mortality) + \beta_8 * \ln(Population\ density) + \beta_9 * \ln(TFR) + \beta_{10} * Percentage\ urban + \beta_{11} * Region + \beta_{12} * Income\ level$$

Negative binomial

$Deaths \sim NBin(r, p)$,

where mean $= \frac{pr}{1-p} =$

$$\beta_0 + \beta_1 * Year\ of\ study +$$
$$\beta_2 * MCV1\ coverage + \beta_3 * Community\ indicator + \beta_4 * Under\ 5\ indicator + \beta_5 * Attack\ rate + \beta_6 * \ln(GDP) +$$
$$\beta_7 * \ln(Under\ 5\ mortality) + \beta_8 * \ln(Population\ density) + \beta_9 * \ln(TFR) + \beta_{10} * Percentage\ urban + \beta_{11} * Region + \beta_{12} * Income\ level$$

and variance $= \frac{pr}{(1-p)^2} =$ variance of the above

Log-linear fitted model selected based on Akaike Information Criterion

$$\ln(CFR) = \beta_0 + \beta_1 * Year\ of\ study + \beta_2 * MCV1\ coverage + \beta_3 * Community\ indicator + \beta_4 * Under\ 5\ indicator + \beta_5 * Attack\ rate + \beta_6 * \ln(Under\ 5\ mortality) + \beta_7 * \ln(Population\ density) + \beta_8 * \ln(TFR) + \beta_9 * Percentage\ urban$$

Table D1. Fitted model definitions and sources

Variable	Type	Definition	Source(s)
CFR	Dependent	The measles case fatality ratio in the specified year as indicated in the selected study.	Appendices N and O
Year of study	Independent	The midpoint year was used in the case of studies across multiple years.	Appendices N and O
MCV1 coverage	Independent	Routine measles immunization first-dose (MCV1) coverage.	WHO (2017)[2]
Community indicator	Independent	A dummy variable used to indicate if a study is in a community-based setting.	Appendices N and O
Under 5 indicator	Independent	A dummy variable used to indicate if a study includes cases and deaths limited to a population under five years of age.	Appendices N and O
Outbreak setting	Independent	A dummy variable used to indicate if a study takes place during a measles outbreak.	Appendices N and O
Attack rate	Independent	Defined as estimated measles incidence divided by the annual birth cohort.	Ferrari (2016)[3]; World Bank (2017)[4]
GNI	Independent	Gross national income per capita in US dollars.	World Bank (2017)[4]
Under 5 mortality	Independent	All-cause under 5 mortality rate per 1000 live births.	World Bank (2017)[4]
Population density	Independent	People per square kilometer of land area.	World Bank (2017)[4]
TFR	Independent	Total fertility rate.	World Bank (2017)[4]
Percentage urban	Independent	Percentage of population in urban areas.	World Bank (2017)[4]
Region	Independent	World Health Organization region.	WHO (2018)[5]
Income level	Independent	World Bank income level.	World Bank (2017)[4]
Educational attainment	Independent	Estimates of average years of educational attainment per capita for people over the age of 15.	IHME (2015)[6]
Urban setting	Independent	A dummy variable used to indicate if a study is in an urban setting.	Appendices N and O
SIA	Independent	A dummy variable used to indicate if a study took place during the same year as supplementary immunization activities.	WHO (2014)[7]
HIV	Independent	Percentage of people aged 15-49 who are infected with HIV.	World Bank (2017)[4]

The fitted model can be used to predict a country-specific CFR with the country-specific inputs from the sources specified in the equations and tables below alongside inputs for year, community-based vs. hospital-based setting indicator, and under 5 vs. over 5 years of age indicator.

Log-linear prediction model

$$\ln(CFR_c) = \beta_0 + \beta_1 * Year + \beta_2 * MCV1\ coverage_c + \beta_3 * Community\ indicator + \beta_4 * Under\ 5\ indicator + \beta_5 * Attack\ rate_c + \beta_6 * \ln(Under\ 5\ mortality_c) + \beta_7 * \ln(Population\ density_c) + \beta_8 * \ln(TFR_c) + \beta_9 * Percentage\ urban_c$$

Log-linear projection model

$$\ln(CFR_c) = \beta_0 + \beta_1 * Year + \beta_2 * MCV1\ coverage_c + \beta_3 * Community\ indicator + \beta_4 * Under\ 5\ indicator + \beta_5 * \ln(Under\ 5\ mortality_c) + \beta_6 * \ln(Population\ density_c) + \beta_7 * \ln(TFR_c) + \beta_8 * Percentage\ urban_c$$

Table D2. Results of the log-linear prediction model selected for 1990-2015 measles CFR predictions in LMICs

	Coefficient	Standard error	P-value
Intercept	5·12	30·14	0·865
Year of study	-0·01	0·02	0·526
Routine first-dose measles immunization (MCV1) coverage	-0·17	0·37	0·642
Community study indicator	-0·67	0·14	<0·001
Under-5 indicator	1·29	0·54	0·019
Attack rate	0·16	0·07	0·026
Log under-5 mortality rate	1·57	0·32	<0·001
Log population density	-0·02	0·07	0·817
Log total fertility rate (TFR)	0·62	0·72	0·394
Percentage urban	4·84	0·75	<0·001

Table D3. Prediction and projection model definitions and sources

Variable	Type	Definition	Source(s)
CFR_c	Dependent	The measles case fatality ratio predicted for a specified year by country.	
Year	Independent	Linear time by year: 1990 – 2015 for the prediction model and 2016 – 2030 for the projection model.	Input
MCV1 $coverage_c$	Independent	Routine measles immunization first-dose (MCV1) coverage.	WHO (2017)[2]
Community indicator	Independent	A dummy variable used to indicate predictions in either a community-based or hospital-based setting.	Input
Under 5 indicator	Independent	A dummy variable used to indicate predictions in either a population under five years of age or a population over five years of age.	Input
Attack $rate_c$*	Independent	Defined as estimated measles incidence divided by the annual birth cohort.	Ferrari (2016)[3]; World Bank (2017)[4]
Under 5 $mortality_c$	Independent	All-cause under 5 mortality rate per 1000 live births.	World Bank (2017)[4]
Population $density_c$	Independent	People per square kilometer of land area.	World Bank (2017)[4]
TFR_c	Independent	Total fertility rate.	World Bank (2017)[4]
Percentage $urban_c$	Independent	Percentage of population in urban areas.	World Bank (2017)[4]

*Not included in projection model.

Appendix E. Community-based studies* included in the measles case fatality ratio (CFR) analysis

Country	Year(s) of study	Midpoint year	Setting	Type of study	Outbreak setting (Y/N)	Under five indicator*	Cases	Deaths	Case fatality ratio	Ref.
Bangladesh	1975	1975	Rural	Analysis of routine surveillance	N	1	510	22	4·31%	[8,9]
Bangladesh	1980	1980	Rural	Cohort study	N	1	3,458	61	1·76%	[10]
Bangladesh	1980	1980	Rural	Analysis of routine surveillance	Y	1	77	1	1·30%	[11]
Bangladesh	1984	1984	Rural	Cohort study	N	1	2,354	41	1·74%	[12]
Bangladesh	1984	1984	Rural	Cohort study	N	0	931	5	0·54%	[12]
Bangladesh	1980-1988	1984	Rural	Analysis of routine surveillance	N	1	2,430	48	1·98%	[13]
Bangladesh	1980-1988	1984	Rural	Analysis of routine surveillance	N	0	1,084	3	0·28%	[13]
Bangladesh	1989	1989	Rural	Analysis of routine surveillance	N	1	3,607	30	0·83%	[14]
Bulgaria	2009	2009	Both	Outbreak investigation	Y	1	24,253	24	0·10%	[15]
Burkina Faso	2000	2000	Urban	Analysis of routine surveillance	N	1	940	29	3·09%	[16]
Burkina Faso	2000	2000	Urban	Analysis of routine surveillance	N	0	2,035	16	0·79%	[16]
Burkina Faso	2009	2009	Both	Outbreak investigation	Y	1	125	4	3·20%	[17]
Burkina Faso	2009	2009	Both	Outbreak investigation	Y	0	301	4	1·33%	[17]
Burundi	1988	1988	Rural	Outbreak investigation	Y	1	357	22	6·16%	[18]
Cambodia	2000	2000	Rural	Outbreak investigation	Y	NA	228	4	1·75%	[19]
Central African Republic	2011	2011	Both	Outbreak investigation	Y	NA	723	2	0·28%	[20]
Chad	1993	1993	Urban	Outbreak investigation	Y	1	824	61	7·40%	[21]
Chad	2004	2004	Urban	Outbreak investigation	Y	1	473	19	4·02%	[22]
China	2005	2005	Both	Analysis of routine surveillance	Y	NA	124,865	55	0·04%	[23]
Congo, Democratic Republic of the	1974-1977	1976	Urban	Cohort study	N	1	1,069	65	6·08%	[8,24]
Congo, Democratic Republic of the	2010	2010	Both	Analysis of routine surveillance	Y	NA	77,241	1,085	1·40%	[25]
Congo, Democratic Republic of the	2011	2011	Both	Outbreak investigation	Y	NA	10,742	403	3·75%	[26]
Ethiopia	1981	1981	Urban	Cohort study	Y	1	63	17	26·98%	[8,27]
Ethiopia	2004-2009	2007	Both	Analysis of routine surveillance	Y	NA	8,044	112	1·39%	[28]
Gambia	1981	1981	Rural	Outbreak investigation	Y	1	77	18	23·38%	[29]

Country	Year(s) of study	Midpoint year	Setting	Type of study	Outbreak setting (Y/N)	Under five indicator*	Cases	Deaths	Case fatality ratio	Ref.
Gambia	1981	1981	Rural	Outbreak investigation	Y	0	57	2	3·51%	29
Gambia	1981	1981	Rural	Outbreak investigation	Y	1	87	8	9·20%	30
Gambia	1981	1981	Rural	Outbreak investigation	Y	0	54	2	3·70%	30
Gambia	1984	1984	Rural	Outbreak investigation	Y	NA	54	0	0·00%	31
Ghana	1989	1989	Rural	Cohort study	N	1	717	132	18·41%	32
Ghana	1989	1989	Rural	Cohort study	N	0	244	19	7·79%	32
Guinea-Bissau	1979	1979	Urban	Outbreak investigation	Y	1	78	14	17·95%	33
Guinea-Bissau	1979	1979	Urban	Outbreak investigation	Y	0	20	1	5·00%	33
Guinea-Bissau	1979	1979	Both	Analysis of routine surveillance	N	1	356	74	20·79%	34
Guinea-Bissau	1979	1979	Both	Analysis of routine surveillance	N	0	103	3	2·91%	34
Guinea-Bissau	1979-1982	1981	Rural	Cohort study	N	1	101	34	23·66%	35
Guinea-Bissau	1979-1982	1981	Rural	Cohort study	N	0	61	4	6·56%	35
Guinea-Bissau	1980-1982	1981	Urban	Analysis of routine surveillance	Y	1	104	14	13·46%	36
Guinea-Bissau	1980-1982	1981	Urban	Analysis of routine surveillance	Y	0	34	3	8·82%	36
Guinea-Bissau	1980-1982	1981	Urban	Cohort study	N	NA	161	23	14·29%	37
Guinea-Bissau	1980-1984	1982	Both	Analysis of routine surveillance	N	1	367	42	11·44%	34
Guinea-Bissau	1980-1984	1982	Both	Analysis of routine surveillance	N	0	118	6	5·08%	34
Guinea-Bissau	1982-1984	1983	Urban	Analysis of routine surveillance	N	1	114	9	7·89%	38
Guinea-Bissau	1985-1987	1986	Urban	Analysis of routine surveillance	N	1	112	11	9·82%	38
Guinea-Bissau	2003-2004	2003	Urban	Other	Y	1	77	7	9·09%	39
India	1974	1974	Rural	Cohort study	Y	NA	112	4	3·57%	8,40
India	1974	1974	Rural	Cohort study	Y	NA	25	0	0·00%	8,40
India	1976-1978	1977	Rural	Cohort study	N	NA	862	2	0·23%	41,42
India	1977	1977	Rural	Outbreak investigation	Y	1	56	9	16·07%	43
India	1977	1977	Rural	Outbreak investigation	Y	0	9	0	0·00%	43
India	1979	1979	Rural	Outbreak investigation	Y	1	78	8	10·26%	44
India	1974-1986	1980	Rural	Cohort study	N	1	291	8	2·75%	45

Country	Year(s) of study	Midpoint year	Setting	Type of study	Outbreak setting (Y/N)	Under five indicator*	Cases	Deaths	Case fatality ratio	Ref.
India	1974-1986	1980	Rural	Cohort study	N	0	120	1	0·83%	[45]
India	1980	1980	Rural	Analysis of routine surveillance	N	NA	266	9	3·38%	[46,47]
India	1980	1980	Rural	Other	Y	NA	55	Not listed	12·73%	[46]
India	1980	1980	Urban	Cohort study	N	NA	731	10	1·37%	[46,48]
India	1980-1981	1981	Rural	Cohort study	N	1	82	3	3·66%	[49]
India	1982	1982	Rural	Outbreak investigation	Y	NA	113	0	0·00%	[46,50]
India	1982	1982	Rural	Outbreak investigation	Y	1	88	4	4·55%	[46,51]
India	1983	1983	Rural	Cross-sectional study	N	NA	241	2	0·83%	[52]
India	1983	1983	Rural	Analysis of routine surveillance	N	1	132	0	0·00%	[53]
India	1984	1984	Rural	Outbreak investigation	Y	NA	515	14	2·72%	[54]
India	1984	1984	Rural	Analysis of routine surveillance	N	NA	430	7	1·63%	[46,55]
India	1984	1984	Rural	Outbreak investigation	Y	NA	133	19	14·29%	[46,56]
India	1985	1985	Rural	Other	Y	1	2,218	Not listed	3·11%	[46]
India	1985	1985	Rural	Other	Y	1	46	Not listed	23·92%	[46]
India	1985	1985	Urban	Cross-sectional study	N	NA	189	0	0·00%	[46,57]
India	1986	1986	Rural	Outbreak investigation	Y	1	292	47	16·10%	[58]
India	1986	1986	Rural	Outbreak investigation	Y	0	448	28	6·25%	[58]
India	1986	1986	Rural	Cross-sectional study	N	1	97	1	1·03%	[59]
India	1986	1986	Rural	Outbreak investigation	Y	NA	217	11	5·07%	[46,60]
India	1991	1991	Rural	Outbreak investigation	Y	1	44	15	34·09%	[61]
India	1991	1991	Rural	Outbreak investigation	Y	0	4	0	0·00%	[61]
India	1991	1991	Rural	Outbreak investigation	Y	1	113	19	16·81%	[61]
India	1991	1991	Rural	Outbreak investigation	Y	0	15	1	6·67%	[61]
India	1992	1992	Rural	Other	Y	NA	93	Not listed	8·60%	[46]
India	1992	1992	Rural	Outbreak investigation	Y	NA	14,522	600	4·13%	[62]
India	1993	1993	Rural	Outbreak investigation	Y	NA	6,392	152	2·38%	[62]
India	1994	1994	Rural	Outbreak investigation	Y	NA	10,561	258	2·44%	[62]

Country	Year(s) of study	Midpoint year	Setting	Type of study	Outbreak setting (Y/N)	Under five indicator*	Cases	Deaths	Case fatality ratio	Ref.
India	1995	1995	Rural	Outbreak investigation	Y	NA	1,931	72	3·73%	[62]
India	1996	1996	Rural	Outbreak investigation	Y	1	1,160	70	6·03%	[62]
India	1996	1996	Rural	Outbreak investigation	Y	0	819	29	3·54%	[62]
India	1999	1999	Urban	Cross-sectional study	N	1	290	0	0·00%	[63]
India	1999	1999	Rural	Outbreak investigation	Y	NA	70	2	2·86%	[41,64]
India	1999	1999	Both	Outbreak investigation	Y	NA	283	0	0·00%	[65]
India	2003	2003	Urban	Outbreak investigation	Y	NA	12	2	16·67%	[66]
India	2003	2003	Urban	Outbreak investigation	Y	NA	58	0	0·00%	[67]
India	2004	2004	Rural	Outbreak investigation	Y	NA	1,204	14	1·16%	[68]
India	2004	2004	Rural	Outbreak investigation	Y	NA	69	0	0·00%	[41,69]
India	2004-2006	2005	Both	Outbreak investigation	Y	NA	432	6	1·39%	[70]
India	2006	2006	Rural	Outbreak investigation	Y	NA	59	0	0·00%	[41,64]
India	2009-2011	2010	Both	Analysis of routine surveillance	Y	NA	772	2	0·26%	[71]
India	2011-2012	2011	Both	Outbreak investigation	Y	1	1,636	20	1·22%	[72]
India	2011-2012	2011	Both	Outbreak investigation	Y	0	2,034	8	0·39%	[72]
Iran	1990	1990	Both	Outbreak investigation	Y	NA	745	4	0·54%	[73]
Kenya	1985	1985	Rural	Outbreak investigation	Y	1	98	12	12·24%	[74]
Kenya	1985	1985	Rural	Outbreak investigation	Y	0	41	1	2·44%	[74]
Kenya	1987	1987	Rural	Outbreak investigation	Y	1	143	18	12·59%	[75]
Kenya	1987	1987	Rural	Outbreak investigation	Y	0	67	2	2·99%	[75]
Malawi	1996-1998	1997	Both	Analysis of routine surveillance	N	1	237	8	3·38%	[76]
Malawi	1996-1998	1997	Both	Analysis of routine surveillance	N	0	305	5	1·64%	[76]
Malawi	2010	2010	Both	Outbreak investigation	Y	1	54,138	139	0·26%	[77]
Malawi	2010	2010	Both	Outbreak investigation	Y	0	53,620	83	0·15%	[77]
Marshall Islands	1977-1978	1977	Rural	Outbreak investigation	Y	NA	340	2	0·59%	[8,78]
Marshall Islands	2003	2003	Both	Outbreak investigation	Y	1	334	1	0·30%	[79]
Marshall Islands	2003	2003	Both	Outbreak investigation	Y	0	487	2	0·41%	[79]
Mexico	1988	1988	Rural	Outbreak investigation	Y	1	70	7	10·00%	[80]

Country	Year(s) of study	Midpoint year	Setting	Type of study	Outbreak setting (Y/N)	Under five indicator*	Cases	Deaths	Case fatality ratio	Ref.
Mexico	1988	1988	Rural	Outbreak investigation	Y	0	130	2	1·54%	[80]
Mozambique	1993	1993	Urban	Analysis of routine surveillance	N	NA	2,363	33	1·40%	[81]
Mozambique	1998	1998	Urban	Analysis of routine surveillance	N	NA	2,720	4	0·15%	[81]
Mozambique	2001-2004	2003	Rural	Analysis of routine surveillance	Y	1	106	7	6·60%	[82]
Myanmar	1983	1983	Rural	Other	N	1	91	11	12·09%	[83]
Myanmar	1983	1983	Rural	Other	N	0	75	3	4·00%	[83]
Nepal	2004	2004	Both	Cross-sectional study	Y	1	1,941	52	2·68%	[84]
Nepal	2004	2004	Both	Cross-sectional study	Y	0	2,705	12	0·44%	[84]
Nepal	2010	2010	Rural	Outbreak investigation	Y	NA	36	1	2·78%	[85]
Niger	1991	1991	Rural	Outbreak investigation	Y	1	418	76	18·18%	[86]
Niger	1991	1991	Urban	Outbreak investigation	Y	1	242	16	6·61%	[87]
Niger	2003	2003	Urban	Outbreak investigation	Y	1	625	29	4·64%	[22]
Niger	2003	2003	Rural	Outbreak investigation	Y	1	641	77	12·01%	[88]
Niger	2003	2003	Rural	Outbreak investigation	Y	0	304	15	4·93%	[88]
Nigeria	1992	1992	Urban	Outbreak investigation	Y	1	481	16	3·33%	[89]
Nigeria	2004	2004	Rural	Outbreak investigation	Y	1	630	68	10·79%	[22]
Nigeria	2007-2012	2010	Rural	Analysis of routine surveillance	N	NA	1,631	2	0·12%	[90]
Nigeria	2008	2008	Rural	Outbreak investigation	Y	1	16	0	0·00%	[91]
Nigeria	2008	2008	Rural	Outbreak investigation	Y	0	2	0	0·00%	[91]
Pakistan	1990	1990	Rural	Outbreak investigation	Y	1	47	3	6·38%	[92]
Pakistan	1990	1990	Rural	Outbreak investigation	Y	0	57	0	0·00%	[92]
Pakistan	2014	2014	Rural	Outbreak investigation	Y	1	48	4	8·33%	[93]
Pakistan	2014	2014	Rural	Outbreak investigation	Y	0	7	0	0·00%	[93]
Peru	1993	1993	Rural	Outbreak investigation	Y	1	39	4	10·26%	[94]
Peru	1993	1993	Rural	Outbreak investigation	Y	0	109	1	0·92%	[94]
Philippines	1983	1983	Rural	Outbreak investigation	Y	NA	126	8	6·35%	[95]
Senegal	1977	1977	Rural	Outbreak investigation	Y	1	160	43	26·88%	[8,96]
Senegal	1977	1977	Rural	Outbreak investigation	Y	0	300	10	3·33%	[8,96]

Country	Year(s) of study	Midpoint year	Setting	Type of study	Outbreak setting (Y/N)	Under five indicator*	Cases	Deaths	Case fatality ratio	Ref.
Senegal	1983-1986	1985	Rural	Analysis of routine surveillance	N	1	966	93	9·63%	[97]
Senegal	1983-1986	1985	Rural	Analysis of routine surveillance	N	0	534	5	0·94%	[97]
Senegal	1985	1985	Rural	Outbreak investigation	Y	1	44	9	20·45%	[98]
Senegal	1985	1985	Rural	Outbreak investigation	Y	0	22	0	0·00%	[98]
Senegal	1987-1990	1989	Rural	Analysis of routine surveillance	N	1	193	6	3·11%	[99]
Senegal	1987-1990	1989	Rural	Analysis of routine surveillance	N	0	437	4	0·92%	[99]
Senegal	1991-1994	1993	Rural	Analysis of routine surveillance	N	1	201	5	2·49%	[100]
Senegal	1991-1994	1993	Rural	Analysis of routine surveillance	N	0	370	3	0·81%	[100]
Senegal	1994	1994	Rural	Outbreak investigation	Y	NA	209	0	0·00%	[101]
Somalia	1978	1978	Rural	Outbreak investigation	Y	NA	910	9	0·99%	[8,102]
South Africa	1980-1998	1989	Rural	Analysis of routine surveillance	N	NA	10,371	101	0·97%	[103]
South Africa	1980-1998	1989	Rural	Analysis of routine surveillance	N	NA	16,406	260	1·58%	[103]
South Africa	2004	2004	Both	Other	Y	NA	109	7	6·42%	[104]
Sri Lanka	1982	1982	Both	Analysis of routine surveillance	N	1	1,630	19	1·17%	[105]
Sri Lanka	1999	1999	Both	Outbreak investigation	Y	1	605	2	0·33%	[106]
Sri Lanka	1999	1999	Both	Outbreak investigation	Y	0	3,913	3	0·08%	[106]
Sudan	1997-1999	1998	Urban	Cohort study	N	NA	95	9	9·47%	[107]
Sudan	2003	2003	Rural	Outbreak investigation	Y	NA	621	8	1·29%	[108]
Thailand	1984	1984	Rural	Outbreak investigation	Y	1	24	8	33·33%	[109]
Thailand	1984	1984	Rural	Outbreak investigation	Y	0	23	3	13·04%	[109]
Zambia	1980-1981	1980	Urban	Outbreak investigation	Y	1	316	5	1·58%	[8,110]
Zimbabwe	1980-1989	1985	Urban	Cross-sectional study	N	1	350	27	7·71%	[111]
Zimbabwe	1980-1989	1985	Urban	Cross-sectional study	N	0	287	1	0·35%	[111]

Note: CFRs from the Aaby, et al.,[8] Sudfeld, et al.,[41] and Singh, et al.[46] reviews include original source references, if the original source was obtained but did not appear in the literature review search results. CFRs in the original source material were prioritized over the data in the published reviews, where applicable.
*Under 5 indicator is listed as 1 if the CFR is specific to children under 5, 0 if the CFR is specific to individuals ages 5 and older, and NA if the CFR was not disaggregated by age.

Appendix F. Hospital-based studies* included in the measles case fatality ratio (CFR) analysis

Country	Year(s) of study	Midpoint year	Setting	Outbreak setting (Y/N)	Under 5 indicator**	Cases	Deaths	Case fatality ratio	Ref.
Afghanistan	1978-1979	1978	Both	N	1	367	13	3·19%	112
Afghanistan	1978-1979	1978	Both	N	0	40	0	0·00%	112
Afghanistan	1980-1982	1981	Both	N	1	715	82	11·47%	113
Afghanistan	1980-1982	1981	Both	N	0	69	3	4·35%	113
Afghanistan	1983-1985	1984	Both	N	NA	717	104	14·50%	114
Burkina Faso	1986-1987	1986	Both	Y	1	693	202	29·15%	115
China	2000-2009	2005	Urban	N	NA	1,328	4	0·30%	116
Congo, Democratic Republic of the	1986-1987	1986	Rural	Y	NA	175	18	10·29%	117
Egypt	1992-1996	1994	Both	N	1	1,201	13	1·08%	118
Ghana	1973	1973	Both	N	NA	439	75	17·08%	119
Ghana	1974	1974	Both	N	NA	357	52	14·57%	119
Ghana	1975	1975	Both	N	NA	602	115	19·10%	119
Ghana	1976	1976	Both	N	NA	469	70	14·93%	119
Ghana	1977	1977	Both	N	NA	545	94	17·25%	119
Ghana	1973	1978	Both	N	1	4,191	725	17·30%	119
Ghana	1978	1978	Both	N	NA	375	72	19·20%	119
Ghana	1979	1979	Both	N	NA	390	76	19·49%	119
Ghana	1980	1980	Both	N	NA	325	50	15·38%	119
Ghana	1981	1981	Both	N	NA	336	49	14·58%	119
Ghana	1982	1982	Both	N	NA	479	75	15·66%	119
Ghana	1996-2000	1998	Both	N	NA	275	14	5·09%	120
Guinea-Bissau	2003	2003	Urban	Y	1	157	14	8·92%	121
Indonesia	1973-1977	1975	Both	N	1	155	44	28·39%	122
Indonesia	1973-1977	1975	Both	N	0	21	2	9·52%	122
Indonesia	1982	1982	Both	N	NA	107	29	27·10%	123
Indonesia	1983	1983	Both	N	NA	48	13	27·08%	123
Indonesia	1984	1984	Both	N	NA	74	15	20·27%	123
Indonesia	1985	1985	Both	N	NA	41	11	26·83%	123

Country	Year(s) of study	Midpoint year	Setting	Outbreak setting (Y/N)	Under 5 indicator**	Cases	Deaths	Case fatality ratio	Ref.
Indonesia	1986	1986	Both	N	NA	40	3	7·50%	123
Kenya	1982-1985	1984	Urban	N	1	7,063	126	1·78%	124
Kenya	1982-1985	1984	Urban	N	0	384	4	1·04%	124
Kenya	1996-2000	1998	Urban	Y	NA	1,200	Not listed	5·63%	125
Malaysia	1990	1990	Both	N	NA	143	2	1·40%	126
Mauritania	2011	2011	Both	N	NA	36	3	8·33%	127
Mexico	1976-1989	1983	Both	N	1	97	15	15·46%	128
Mozambique	2001-2004	2003	Rural	Y	NA	246	8	3·25%	82
Myanmar	1989	1989	Both	N	1	107	21	19·63%	129
Myanmar	1989	1989	Both	N	0	61	5	8·20%	129
Nigeria	1978-1986	1982	Both	N	NA	1,078	Not listed	32·60%	130
Nigeria	1982-1984	1983	Urban	N	1	598	71	11·87%	131
Nigeria	1983-1986	1985	Both	N	1	267	70	26·22%	132
Nigeria	1983-1986	1985	Both	N	0	2	0	0·00%	132
Nigeria	1984-1987	1986	Urban	N	1	424	80	18·87%	133
Nigeria	1984-1987	1986	Urban	N	0	12	0	0·00%	133
Nigeria	1992-1996	1994	Urban	N	1	31	0	0·00%	134
Nigeria	1992-1996	1994	Urban	N	0	5	1	20·00%	134
Nigeria	1994-2004	1999	Urban	N	1	56	4	7·14%	135
Nigeria	2000-2004	2002	Both	N	NA	666	56	8·41%	136
Pakistan	2003-2004	2003	Urban	N	1	42	4	9·52%	137
Pakistan	2003-2005	2004	Rural	N	1	85	6	5·15%	138
Pakistan	2003-2005	2004	Rural	N	0	51	1	5·15%	138
Papua New Guinea	1999-2000	1999	Both	Y	NA	238	10	4·20%	139
Papua New Guinea	2001	2001	Urban	Y	1	417	17	4·08%	140
Papua New Guinea	2001	2001	Urban	Y	0	65	2	3·08%	140
Philippines	1993-1996	1995	Urban	N	NA	180	8	4·44%	141
South Africa	1992-1996	1994	Urban	N	NA	1,647	11	0·67%	103
South Africa	1992-1996	1994	Urban	N	NA	736	23	3·13%	103

Country	Year(s) of study	Midpoint year	Setting	Outbreak setting (Y/N)	Under 5 indicator**	Cases	Deaths	Case fatality ratio	Ref.
South Africa	1997-1999	1998	Urban	N	NA	60	0	0·00%	[103]
South Africa	1997-1999	1998	Urban	N	NA	29	0	0·00%	[103]
South Africa	2009	2009	Urban	Y	1	552	18	3·26%	[142]
South Africa	2010	2010	Urban	N	1	58	18	31·03%	[143]
South Sudan	1985	1985	Urban	N	1	208	48	23·08%	[144]
Tanzania	1981-1983	1982	Both	Y	1	913	72	7·89%	[145]
Zambia	1992-1993	1992	Urban	N	1	917	123	13·41%	[146]
Zambia	1992-1993	1992	Urban	N	0	149	11	7·38%	[146]
Zimbabwe	1987-1989	1988	Both	N	1	Not listed	Not listed	3·30%	[147]
Zimbabwe	1987-1989	1988	Both	N	0	Not listed	Not listed	1·50%	[147]
Zimbabwe	1988	1988	Urban	Y	NA	1,399	20	1·43%	[148]

*All studies were cross-sectional studies of hospital admissions.
**Under 5 indicator is listed as 1 if the CFR is specific to children under 5, 0 if the CFR is specific to individuals ages 5 and older, and NA if the CFR was not disaggregated by age.

Figure G. Histogram of overall measles case fatality ratios (CFRs), extracted from the 1980-2016 review of the literature.

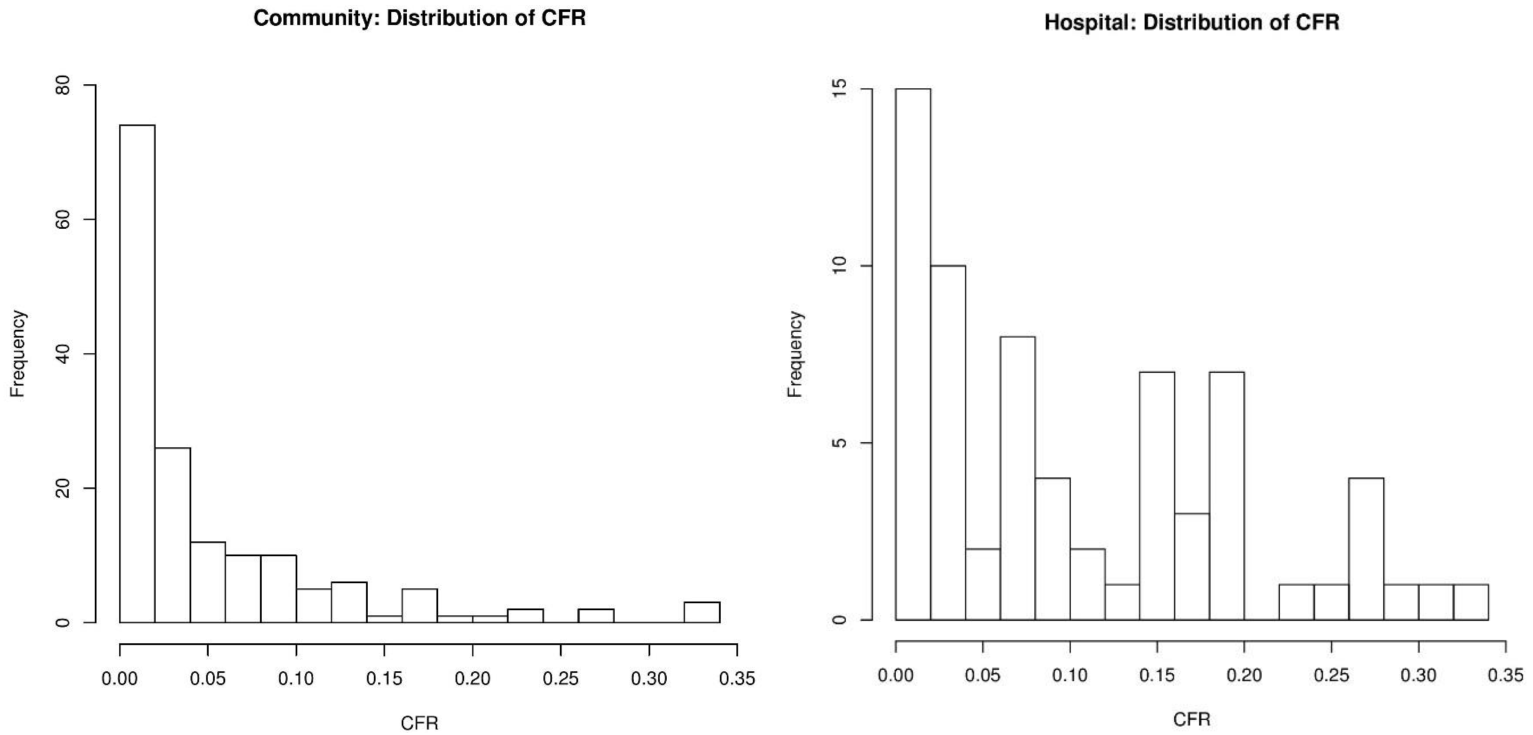

Community-based studies: 158 observations. Hospital-based studies: 68 observations.

Figure H. Measles case fatality ratio (CFR) by country group income level and year of study, extracted from the 1980-2016 review of the literature.

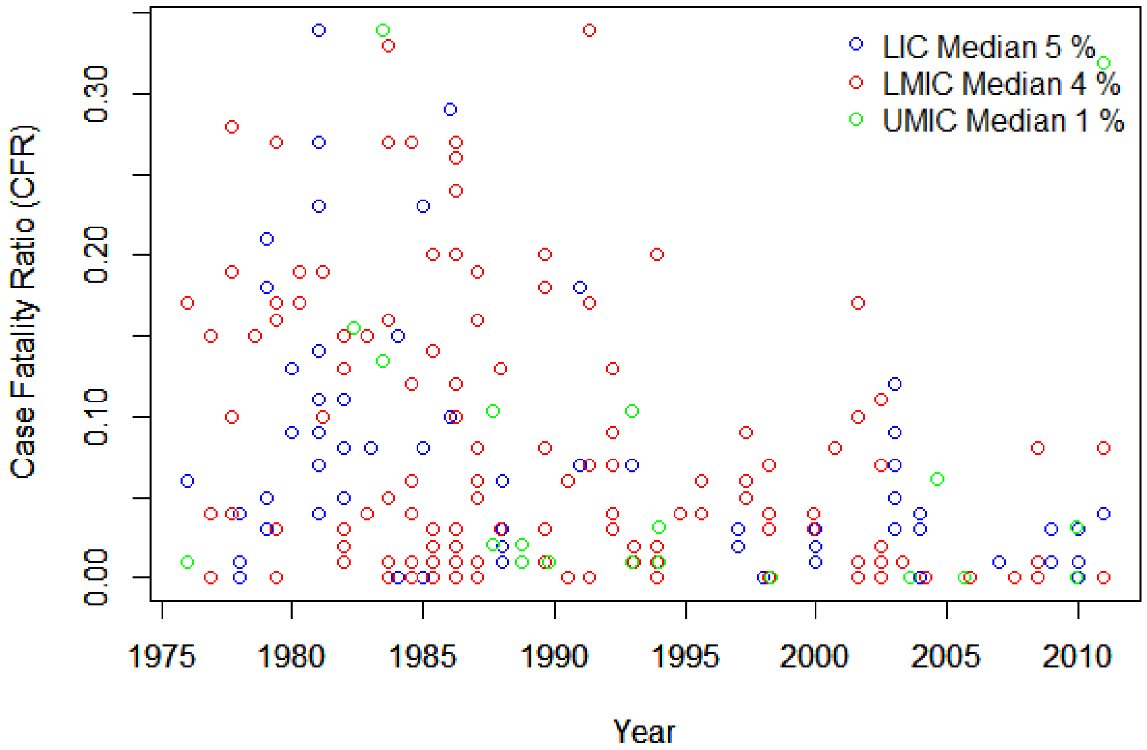

Note: LIC=Low-income country; LMIC=Lower middle-income country; UMIC=Upper middle-income country.
LIC: 65 observations in blue. LMIC: 137 observations in red. UMIC: 24 observations in green.

Figure I. Mean predicted* measles case fatality ratios (CFRs) in each year when the CFR estimate from each study from the literature review is unweighted in the log-linear model estimation.

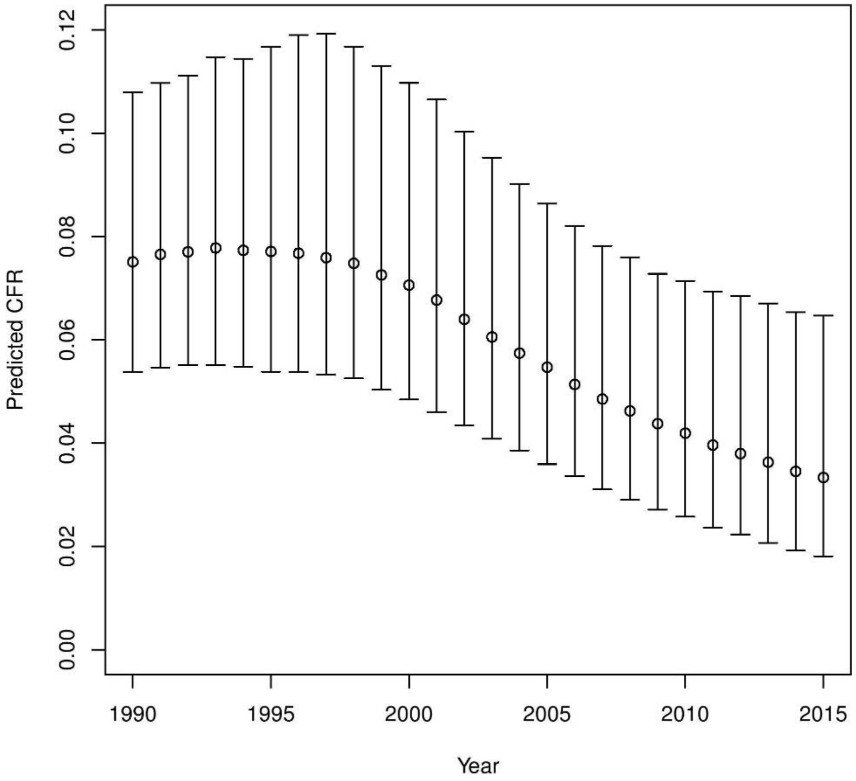

Note: *Predicted CFRs (dots) are averaged arithmetically without weighting across all 136 low- and middle-income countries for all ages in each year in order to provide the mean predicted CFR in that year.

Figure J. Mean predicted* measles case fatality ratios (CFRs) in each year: population-averaged over 136 low- and middle-income countries, all ages.

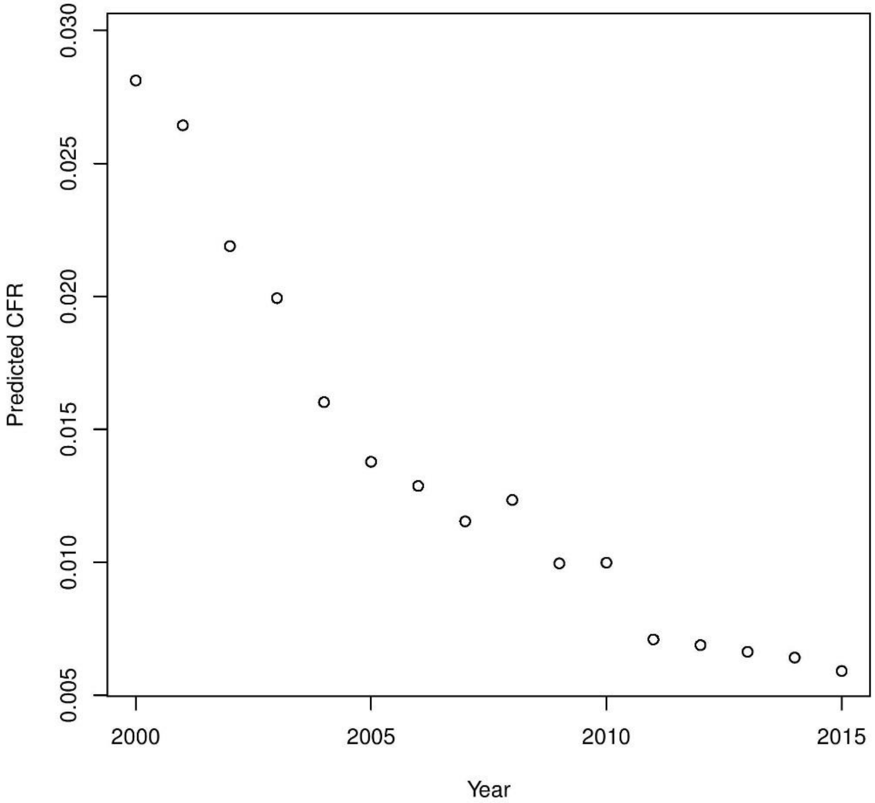

Note: *Predicted CFRs (dots) are a weighted average according to total country-level population across all 136 low- and middle-income countries for all ages in each year in order to provide the mean predicted CFR in that year.

Figure K. Boxplot comparing original* to predicted measles case fatality ratios (CFRs) (below or above age 5)**

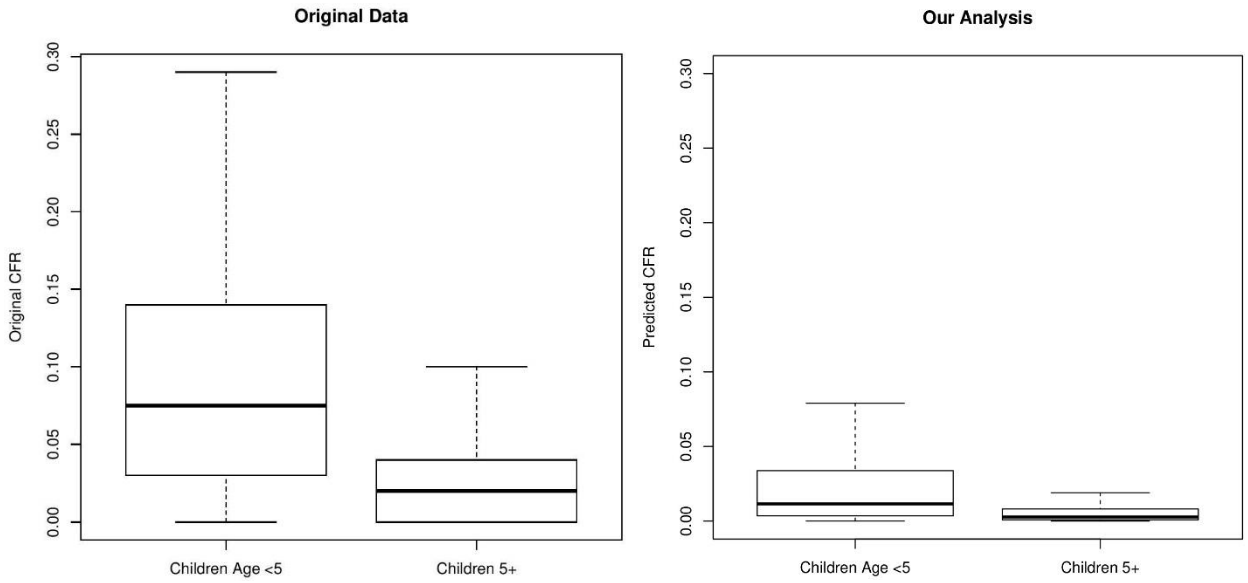

*Original data from our 1980-2016 literature review, including 43 countries.
**Predicted estimates for all 136 low- and middle-income countries over 1990-2015.

Table L. Information loss between 2016–2030 projection and 1990–2015 prediction models

	AIC
**Prediction model**	-337
**Projection model**	-323

AIC: Akaike information criterion.

Note: Prediction model is the log-linear model selected for 1990-2015 covariates (Appendix D); projection model is the same log-linear model for the same covariates in 2016-2030 but without the "attack rate" covariate.

Figure M. Boxplot of projected measles case fatality ratios (CFRs) from 2016-2030 by under-5 mortality rate category for 136 low- and middle-income countries, all ages.

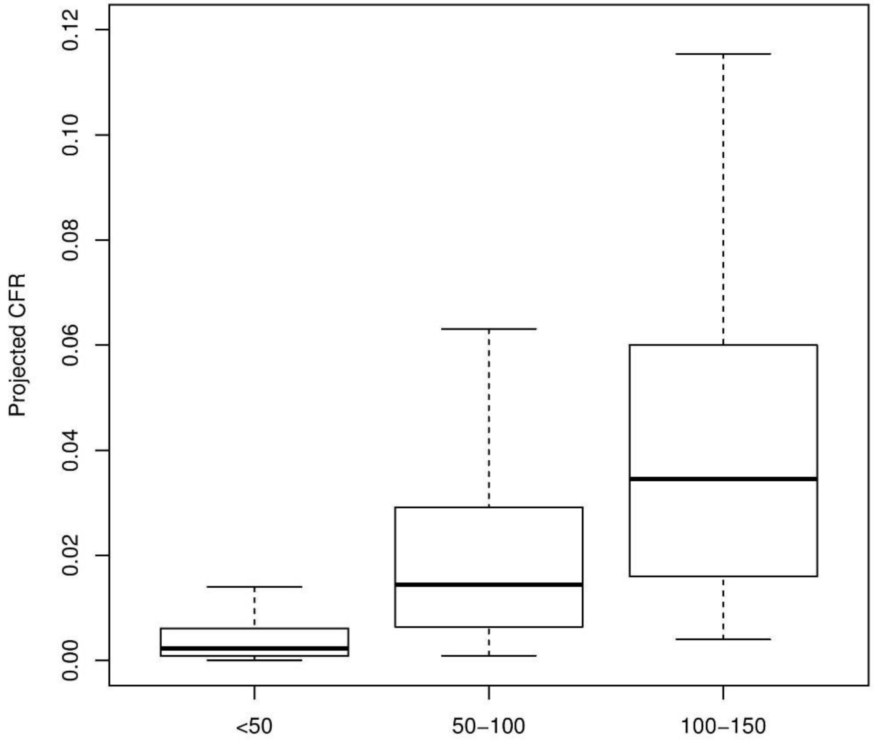

Note: Under-5 mortality rate defined as the estimated number of deaths before age 5 per 1,000 live births.

Table N. Comparison to the findings of Wolfson and colleagues' review of measles case fatality ratios

	Wolfson and colleagues[1] review 1980–2008	Our review 1980–2016		Our analysis 1990–2015	
	Community-Based	Community-Based	Hospital-Based	Community-Based	Hospital-Based
Mean	7·4% (Range: 0·0–40·2%)	5·4% (IQR: 1·0–7·8%)	10·8% (IQR: 3·0–17·0%)	1·5% (CI: 0·5–3·1%)	2·9% (CI: 0·9–6·0%)
Median	3·9%	3·0%	8·0%	1·5%	2·9%

IQR: Interquartile range; CI: 95% confidence interval.

References

1. Wolfson LJ, Grais RF, Luquero FJ, Birmingham ME, Strebel PM. Estimates of measles case fatality ratios: a comprehensive review of community-based studies. International journal of epidemiology. 2009 Feb; 38(1): 192-205.
2. World Health Organization. WHO/UNICEF coverage estimates for 1980-2015. Geneva: WHO/UNICEF. Last updated: 25 July 2017. [Online] Accessed 18 December 2017. Available at: http://www.who.int/immunization/monitoring_surveillance/routine/coverage/en/index4.html. 2017.
3. Ferrari M. Pennsylvania State University and the World Health Organization dynamic measles model incidence and uncertainty bounds. Personal communication, 3 November 2016. 2016.
4. World Bank. World development indicators. Washington, DC: The World Bank; 2017. Last updated: 15 September 2017. [Online] Accessed 18 December 2017. Available at: http://data.worldbank.org/. 2017.
5. World Health Organization. Alphabetical List of WHO Member States. Geneva: WHO/UNICEF. [Online] Available at: http://www.who.int/choice/demography/by_country/en/. 2018.
6. Institute for Health Metrics and Evaluation (IHME). Global Educational Attainment 1970-2015. Seattle, United States: Institute for Health Metrics and Evaluation (IHME), 2015. [Online] Accessed 1 November 2016. Available at: http://ghdx.healthdata.org/record/global-educational-attainment-1970-2015.
7. World Health Organization. Retrospective Measles Data on Supplementary Immunization Activities 2000-2014. Data source: WHO/IVB Database, last updated 30 October 2014. Immunization Vaccines and Biologicals (IVB), World Health Organization.
8. Aaby P. Malnutrition and overcrowding/intensive exposure in severe measles infection: review of community studies. Reviews of infectious diseases. 1988 Mar-Apr; 10(2): 478-91.
9. Koster FT, Curlin GC, Aziz KM, Haque A. Synergistic impact of measles and diarrhoea on nutrition and mortality in Bangladesh. Bulletin of the World Health Organization. 1981; 59(6): 901-8.
10. Bhuiya A, Wojtyniak B, D'Souza S, Nahar L, Shaikh K. Measles case fatality among the under-fives: a multivariate analysis of risk factors in a rural area of Bangladesh. Social science & medicine (1982). 1987; 24(5): 439-43.
11. Shahid NS, Clauquin P, Shaikh K, Zimicki S. Long-term complication of measles in rural Bangladesh. The Journal of tropical medicine and hygiene. 1983 Apr; 86(2): 77-80.
12. Expanded Programme on Immunization. Public health importance of measles: Bangladesh. Wkly Epidemiol Rec. 1986; 61(12): 89-90.
13. Fauveau V, Chakraborty J, Sarder AM, Khan MA, Koenig MA. Measles among under-9-month-olds in rural Bangladesh: its significance for age at immunization. Bulletin of the World Health Organization. 1991; 69(1): 67-72.
14. De Francisco A, Fauveau V, Sarder AM, Chowdhury HR, Chakraborty J, Yunus MD. Measles in rural Bangladesh: issues of validation and age distribution. Int J Epidemiol. 1994 Apr; 23(2): 393-9.

15. Lim TA, Marinova L, Kojouharova M, Tsolova S, Semenza JC. Measles outbreak in Bulgaria: poor maternal educational attainment as a risk factor for medical complications. European journal of public health. 2013 Aug; 23(4): 663-9.
16. Kambire C, Konde MK, Yameogo A, et al. Measles incidence before and after mass vaccination campaigns in Burkina Faso. The Journal of infectious diseases. 2003 May 15; 187 Suppl 1: S80-5.
17. Kidd S, Ouedraogo B, Kambire C, et al. Measles outbreak in Burkina Faso, 2009: a case-control study to determine risk factors and estimate vaccine effectiveness. Vaccine. 2012 Jul 13; 30(33): 5000-8.
18. Chen RT, Weierbach R, Bisoffi Z, et al. A 'post-honeymoon period' measles outbreak in Muyinga sector, Burundi. Int J Epidemiol. 1994 Feb; 23(1): 185-93.
19. Oum S, Chandramohan D, Cairncross S. Community-based surveillance: a pilot study from rural Cambodia. Tropical medicine & international health : TM & IH. 2005 Jul; 10(7): 689-97.
20. Tricou V, Pagonendji M, Manengu C, Mutombo J, Mabo RO, Gouandjika-Vasilache I. Measles outbreak in Northern Central African Republic 3 years after the last national immunization campaign. BMC infectious diseases. 2013; 13: 103.
21. Ndikuyeze A, Cook A, Cutts FT, Bennett S. Priorities in global measles control: report of an outbreak in N'Djamena, Chad. Epidemiology and infection. 1995 Oct; 115(2): 309-14.
22. Grais RF, Dubray C, Gerstl S, et al. Unacceptably high mortality related to measles epidemics in Niger, Nigeria, and Chad. PLoS medicine. 2007 Jan; 4(1): e16.
23. Ji Y, Zhang Y, Xu S, et al. Measles resurgence associated with continued circulation of genotype H1 viruses in China, 2005. Virology journal. 2009; 6: 135.
24. The Kasongo Project Team. Influence of measles vaccination on survival pattern of 7--35-month-old children in Kasongo, Zaire. Lancet (London, England). 1981 Apr 04; 1(8223): 764-7.
25. Grout L, Minetti A, Hurtado N, et al. Measles in Democratic Republic of Congo: an outbreak description from Katanga, 2010-2011. BMC infectious diseases. 2013; 13: 232.
26. N'Goran A A, Ilunga N, Coldiron ME, Grais RF, Porten K. Community-based measles mortality surveillance in two districts of Katanga Province, Democratic Republic of Congo. BMC research notes. 2013; 6: 537.
27. Lindtjorn B. Severe measles in the Gardulla area of southwest Ethiopia. Journal of tropical pediatrics. 1986 Oct; 32(5): 234-9.
28. Mitiku K, Bedada T, Masresha BG, et al. Progress in measles mortality reduction in Ethiopia, 2002-2009. The Journal of infectious diseases. 2011 Jul; 204 Suppl 1: S232-8.
29. Hull HF, Williams PJ, Oldfield F. Measles mortality and vaccine efficacy in rural West Africa. Lancet (London, England). 1983 Apr 30; 1(8331): 972-5.
30. Williams PJ. Effect of measles immunization on child mortality in rural Gambia. Journal of biosocial science Supplement. 1989; 10: 95-104.
31. Lamb WH. Epidemic measles in a highly immunized rural West African (Gambian) village. Rev Infect Dis. 1988 Mar-Apr; 10(2): 457-62.
32. Dollimore N, Cutts F, Binka FN, Ross DA, Morris SS, Smith PG. Measles incidence, case fatality, and delayed mortality in children with or without vitamin A supplementation in rural Ghana. American journal of epidemiology. 1997 Oct 15; 146(8): 646-54.

33. Aaby P, Bukh J, Lisse IM, Smits AJ. Measles mortality, state of nutrition, and family structure: a community study from Guinea-Bissau. The Journal of infectious diseases. 1983 Apr; 147(4): 693-701.
34. Aaby P, Bukh J, Lisse IM, da Silva MC. Decline in measles mortality: nutrition, age at infection, or exposure? British medical journal (Clinical research ed). 1988 Apr 30; 296(6631): 1225-8.
35. Aaby P, Bukh J, Lisse IM, et al. Determinants of measles mortality in a rural area of Guinea-Bissau: crowding, age, and malnutrition. Journal of tropical pediatrics. 1984 Jun; 30(3): 164-8.
36. Aaby P, Bukh J, Lisse IM, da Silva MC. Further community studies on the role of overcrowding and intensive exposure on measles mortality. Rev Infect Dis. 1988 Mar-Apr; 10(2): 474-7.
37. Aaby P, Bukh J, Lisse IM, Smits AJ. Introduction of measles into a highly immunised West African community: the role of health care institutions. Journal of epidemiology and community health. 1985 Jun; 39(2): 113-6.
38. Aaby P, Knudsen K, Jensen TG, et al. Measles incidence, vaccine efficacy, and mortality in two urban African areas with high vaccination coverage. The Journal of infectious diseases. 1990 Nov; 162(5): 1043-8.
39. Martins CL, Garly ML, Bale C, et al. Protective efficacy of standard Edmonston-Zagreb measles vaccination in infants aged 4.5 months: interim analysis of a randomised clinical trial. British medical journal (Clinical research ed). 2008; 337: a661.
40. Agarwal DK, Dutta A, Arora RR, Nair MRV. Natural history of measles in rural and urban community of Varanasi. J Commun Dis. 1976; 8: 289-98.
41. Sudfeld CR, Halsey NA. Measles case fatality ratio in India a review of community based studies. Indian pediatrics. 2009 Nov; 46(11): 983-9.
42. Garai R, Chakraborty AK. Measles in a rural community. Indian journal of public health. 1981 Jul-Sep; 24(3): 150-3.
43. John TJ, Joseph A, George TI, Radhakrishnan J, Singh RP, George K. Epidemiology and prevention of measles in rural south India. The Indian journal of medical research. 1980 Aug; 72: 153-8.
44. Cherian T, Joseph A, John TJ. Low antibody response in infants with measles and children with subclinical measles virus infection. The Journal of tropical medicine and hygiene. 1984 Feb; 87(1): 27-31.
45. Chand P, Rai RN, Chawla U, Tripathi KC, Datta KK. Epidemiology of measles--a thirteen years prospective study in a village. J Commun Dis. 1989 Sep; 21(3): 190-9.
46. Singh J, Sharma RS, Verghese T. Measles mortality in India: a review of community based studies. The Journal of communicable diseases. 1994 Dec; 26(4): 203-14.
47. Vasudev JP, Nandan D, Chandra R, Srivastava BC. Post measles complications in a rural population. J Commun Dis. 1983 Dec; 15(4): 249-52.
48. Swami SS, Chandra S, Dudani IU, Sharma R, Mathur MM. Epidemiology of measles in western Rajasthan. J Commun Dis. 1987 Dec; 19(4): 370-2.
49. Dhanoa J, Cowan B. Measles in the community--a study in non-hospitalised young children in Punjab. Journal of tropical pediatrics. 1982 Apr; 28(2): 59-61.
50. Jajoo UN, Chhabra S, Gupta OP, Jain AP. Measles epidemic in a rural community near Sevagram (Vidarbha). Indian journal of public health. 1984 Oct-Dec; 28(4): 204-7.

51. Sharma RS, Kaushic VK, Johri SP, Ray SN. An epidemiological investigation of measles outbreak in Alwar--Rajasthan. J Commun Dis. 1984 Dec; 16(4): 299-303.
52. Lakhanpal U, Rathore MS. Epidemiology of measles in rural area of Punjab. J Commun Dis. 1986 Sep; 18(3): 185-8.
53. Phaneendra Rao RS, Kumari J, Krishna Rao TS, Narasimham VL. Measles in a rural community. J Commun Dis. 1988 Jun; 20(2): 131-5.
54. Bhatia R. Measles outbreak in village Tophema in Nagaland. J Commun Dis. 1985 Jun; 17(2): 185-9.
55. Lobo J, Reddaiah VP, Kapoor SK, Nath LM. Epidemiology of measles in a rural community. Indian journal of pediatrics. 1987 Mar-Apr; 54(2): 261-5.
56. Sharma RS. An epidemiological study of measles epidemic in district Bhilwara, Rajasthan. J Commun Dis. 1988 Dec; 20(4): 301-11.
57. Mangal N, Shah K, Sitaraman S. Epidemiological study of measles in urban (slum) area of Jaipur. Indian Pediatr. 1990 Nov; 27(11): 1216-7.
58. Narain JP, Khare S, Rana SR, Banerjee KB. Epidemic measles in an isolated unvaccinated population, India. Int J Epidemiol. 1989 Dec; 18(4): 952-8.
59. Satpathy SK, Chakraborty AK. Epidemiological study of measles in Singur, West Bengal. J Commun Dis. 1990 Mar; 22(1): 23-6.
60. Gupta BP, Swami HM, Bhardwaj AK, Vaidya NK, Sharma CD, Kaushal RK. An outbreak of measles in a remote tribal area of Himachal Pradesh. Indian J Comm Health. 1989; 5(1): 25-8.
61. Risbud AR, Prasad SR, Mehendale SM, et al. Measles outbreak in a tribal population of Thane district, Maharashtra. Indian Pediatr. 1994 May; 31(5): 543-51.
62. Singh J, Kumar A, Rai RN, et al. Widespread outbreaks of measles in rural Uttar Pradesh, India, 1996: high risk areas and groups. Indian Pediatr. 1999 Mar; 36(3): 249-56.
63. Ray SK, Mallik S, Munsi AK, Mitra SP, Baur B, Kumar S. Epidemiological study of measles in slum areas of Kolkata. Indian journal of pediatrics. 2004 Jul; 71(7): 583-6.
64. John S, Sanghi S, Prasad S, Bose A, George K. Two doses of measles vaccine: are some states in India ready for it? Journal of tropical pediatrics. 2009 Aug; 55(4): 253-6.
65. Thakur JS, Ratho RK, Bhatia SP, et al. Measles outbreak in a Periurban area of Chandigarh: need for improving vaccine coverage and strengthening surveillance. Indian journal of pediatrics. 2002 Jan; 69(1): 33-7.
66. Ratho RK, Mishra B, Singh T, Rao P, Kumar R. Measles outbreak in a migrant population. Indian journal of pediatrics. 2005 Oct; 72(10): 893-4.
67. Sharma MK, Bhatia V, Swami HM. Outbreak of measles amongst vaccinated children in a slum of Chandigarh. Indian journal of medical sciences. 2004 Feb; 58(2): 47-53.
68. Mishra A, Mishra S, Jain P, Bhadoriya RS, Mishra R, Lahariya C. Measles related complications and the role of vitamin A supplementation. Indian journal of pediatrics. 2008 Sep; 75(9): 887-90.
69. Gupta BP, Sharma S. Measles outbreak in a rural area near Shimla. Indian Journal of Community Medicine. 2006; 31(2): 106-8.
70. Murhekar MV, Hutin YJ, Ramakrishnan R, et al. The heterogeneity of measles epidemiology in India: implications for improving control measures. The Journal of infectious diseases. 2011 Jul; 204 Suppl 1: S421-6.

71. Bose AS, Jafari H, Sosler S, et al. Case based measles surveillance in Pune: evidence to guide current and future measles control and elimination efforts in India. PloS one. 2014; 9(10): e108786.
72. Murhekar MV, Ahmad M, Shukla H, et al. Measles case fatality rate in Bihar, India, 2011-12. PloS one. 2014; 9(5): e96668.
73. Janghorbani M, Daie Parizi MH, Ghorbani K. Measles epidemics in Kerman City, Iran. Public health. 1993 Mar; 107(2): 79-87.
74. Burstrom B, Aaby P, Mutie DM, Kimani G, Bjerregaard P. Severe measles outbreak in western Kenya. East African medical journal. 1992 Aug; 69(8): 419-23.
75. Burstrom B, Aaby P, Mutie DM. Child mortality impact of a measles outbreak in a partially vaccinated rural African community. Scandinavian journal of infectious diseases. 1993; 25(6): 763-9.
76. Yamaguchi S, Dunga A, Broadhead RL, Brabin BJ. Epidemiology of measles in Blantyre, Malawi: analyses of passive surveillance data from 1996 to 1998. Epidemiology and infection. 2002 Oct; 129(2): 361-9.
77. Minetti A, Kagoli M, Katsulukuta A, et al. Lessons and challenges for measles control from unexpected large outbreak, Malawi. Emerging infectious diseases. 2013 Feb; 19(2): 202-9.
78. McIntyre RC, Preblud SR, Polloi A, Korean M. Measles and measles vaccine efficacy in a remote island population. Bulletin of the World Health Organization. 1982; 60(5): 767-75.
79. Hyde TB, Dayan GH, Langidrik JR, et al. Measles outbreak in the Republic of the Marshall Islands, 2003. Int J Epidemiol. 2006 Apr; 35(2): 299-306.
80. Sanchez-Vargas MC, Lopez-Ortiz O, Bustamante-Hernandez A. [Measles outbreak in the indian community of Yaitepec, Oaxaca, 1988]. Boletin medico del Hospital Infantil de Mexico. 1990 Jul; 47(7): 489-94. Brote de sarampion en la comunidad indigena de Yaitepec, Oaxaca, 1988.
81. Cliff J, Simango A, Augusto O, Van Der Paal L, Biellik R. Failure of targeted urban supplemental measles vaccination campaigns (1997-1999) to prevent measles epidemics in Mozambique (1998-2001). The Journal of infectious diseases. 2003 May 15; 187 Suppl 1: S51-7.
82. Mandomando I, Naniche D, Pasetti MF, et al. Assessment of the epidemiology and burden of measles in Southern Mozambique. The American journal of tropical medicine and hygiene. 2011 Jul; 85(1): 146-51.
83. Chin J, Thaung UM. The unchanging epidemiology and toll of measles in Burma. Bulletin of the World Health Organization. 1985; 63(3): 551-8.
84. Joshi AB, Luman ET, Nandy R, Subedi BK, Liyanage JB, Wierzba TF. Measles deaths in Nepal: estimating the national case-fatality ratio. Bulletin of the World Health Organization. 2009 Jun; 87(6): 456-65.
85. Sitaula S, Awasthi GR, Thapa JB, Joshi KP, Ramaiya A. Measles outbreak among unvaccinated children in Bajura. JNMA; journal of the Nepal Medical Association. 2010 Oct-Dec; 50(180): 273-6.
86. Expanded programme on immunization. High measles case-fatality rates during an outbreak in a rural area. Niger. Wkly Epidemiol Rec. 1993 May 14; 68(20): 142-5.
87. Malfait P, Jataou IM, Jollet MC, Margot A, De Benoist AC, Moren A. Measles epidemic in the urban community of Niamey: transmission patterns, vaccine efficacy and immunization

strategies, Niger, 1990 to 1991. The Pediatric infectious disease journal. 1994 Jan; 13(1): 38-45.
88. Nandy R, Handzel T, Zaneidou M, et al. Case-fatality rate during a measles outbreak in eastern Niger in 2003. Clinical infectious diseases : an official publication of the Infectious Diseases Society of America. 2006 Feb 1; 42(3): 322-8.
89. Byass P, Adedeji MD, Mongdem JG, Zwandor AC, Brew-Graves SH, Clements CJ. Assessment and possible control of endemic measles in urban Nigeria. Journal of public health medicine. 1995 Jun; 17(2): 140-5.
90. Fatiregun AA, Adebowale AS, Fagbamigbe AF. Epidemiology of measles in Southwest Nigeria: an analysis of measles case-based surveillance data from 2007 to 2012. Transactions of the Royal Society of Tropical Medicine and Hygiene. 2014 Mar; 108(3): 133-40.
91. Fatiregun AA, Olowookere SA, Abubakar O, Aderibigbe A. Small-scale outbreak of measles in the Irewole local government area of Osun State in Nigeria. Asian Pacific Journal of Tropical Medicine. 2009; 2: 33-6.
92. Murray M, Rasmussen Z. Measles outbreak in a northern Pakistani village: epidemiology and vaccine effectiveness. American journal of epidemiology. 2000 Apr 15; 151(8): 811-9.
93. Saeed A, Butt ZA, Malik T. Investigation of Measles Outbreak in a District of Balochistan Province, Pakistan. Journal of Ayub Medical College, Abbottabad : JAMC. 2015 Oct-Dec; 27(4): 900-3.
94. Sniadack DH, Moscoso B, Aguilar R, Heath J, Bellini W, Chiu MC. Measles epidemiology and outbreak response immunization in a rural community in Peru. Bulletin of the World Health Organization. 1999; 77(7): 545-52.
95. Almoradie-Javonillo I, Javonillo TO. Profile of a measles epidemic in a remote Philippine barrio. Journal of the Philippine Medical Association. 1984; 60(3): 103-8.
96. Pison G. Dynamique d'une population traditionelle: les Peul Bande (Senegal oriental). Institut national d'etudes demographiques. Cahier no. 99. Paris: Presses Universitaires de France, 1982. 1982.
97. Aaby P. Influence of cross-sex transmission on measles mortality in rural Senegal. Lancet (London, England). 1992 Aug 15; 340(8816): 388-91.
98. Pison G, Bonneuil N. Increased risk of measles mortality for children with siblings among the Fula Bande, Senegal. Rev Infect Dis. 1988 Mar-Apr; 10(2): 468-70.
99. Samb B, Aaby P, Whittle H, Seck AM, Simondon F. Decline in measles case fatality ratio after the introduction of measles immunization in rural Senegal. American journal of epidemiology. 1997 Jan 01; 145(1): 51-7.
100. Aaby P, Whittle H, Cisse B, Samb B, Jensen H, Simondon F. The frailty hypothesis revisited: mainly weak children die of measles. Vaccine. 2001 Dec 12; 20(5-6): 949-53.
101. Cisse B, Aaby P, Simondon F, Samb B, Soumare M, Whittle H. Role of schools in the transmission of measles in rural Senegal: implications for measles control in developing countries. American journal of epidemiology. 1999 Feb 15; 149(4): 295-301.
102. Epidemiology of measles in a rural community. Wkly Epidemiol Rec. 1980; 55(12): 85-7.
103. Uzicanin A, Eggers R, Webb E, et al. Impact of the 1996-1997 supplementary measles vaccination campaigns in South Africa. International journal of epidemiology. 2002 Oct; 31(5): 968-76.

104. McMorrow ML, Gebremedhin G, van den Heever J, et al. Measles outbreak in South Africa, 2003-2005. South African medical journal = Suid-Afrikaanse tydskrif vir geneeskunde. 2009 May; 99(5): 314-9.
105. Expanded Programme on Immunization. Public health importance of measles: Sri Lanka. Wkly Epidemiol Rec. 1985; 60(13): 95-7.
106. Puvimanasinghe JP, Arambepola CK, Abeysinghe NM, Rajapaksa LC, Kulatilaka TA. Measles outbreak in Sri Lanka, 1999-2000. The Journal of infectious diseases. 2003 May 15; 187 Suppl 1: S241-5.
107. Ibrahim SA, Mustafa OM, Mukhtar MM, et al. Measles in suburban Khartoum: an epidemiological and clinical study. Tropical medicine & international health : TM & IH. 2002 May; 7(5): 442-9.
108. Coronado F, Musa N, El Tayeb el SA, et al. Retrospective measles outbreak investigation: Sudan, 2004. Journal of tropical pediatrics. 2006 Oct; 52(5): 329-34.
109. Expanded Programme on Immunization. Measles outbreak among the hill tribes. Wkly Epidemiol Rec. 1985; 60(11): 79.
110. Rolfe M. Measles immunization in the Zambian copperbelt: cause for concern. Transactions of the Royal Society of Tropical Medicine and Hygiene. 1982; 76(4): 529-30.
111. Marufu T, Siziya S, Tshimanga M, Murugasampillay S, Mason E, Manyame B. Factors associated with measles complications in Gweru, Zimbabwe. East African medical journal. 2001 Mar; 78(3): 135-8.
112. Arya LS, Azamy S, Ghani AR, Singh M. Outcome of measles in Afghanistan. Indian Pediatr. 1981 Feb; 18(2): 112-6.
113. Arya LS, Taana I, Tahiri C, Saidali A, Singh M. Spectrum of complications of measles in Afghanistan: a study of 784 cases. The Journal of tropical medicine and hygiene. 1987 Jun; 90(3): 117-22.
114. Choudhry VP, Atmar M, Amin I, Aram GN, Ghani R. Effect of protein energy malnutrition on the immediate outcome of measles. Indian journal of pediatrics. 1987 Sep-Oct; 54(5): 717-22.
115. Sahuguede P, Roisin A, Sanou I, Nacro B, Tall F. [An epidemic of measles in Burkina Faso: 714 cases hospitalized in the hospital at Bobo-Dioulasso. A study of risk factors]. Annales de pediatrie. 1989 Apr; 36(4): 244-51. Epidemie de rougeole au Burkina Faso: 714 cas hospitalises a l'hopital de Bobo-Dioulasso. Etude des facteurs de risque.
116. Ye Y, Wang W, Wang X, Yu H. The clinical epidemiology of pediatric patients with measles from 2000 to 2009 in shanghai, china. Clinical pediatrics. 2011 Oct; 50(10): 916-22.
117. Fischer PR. Measles in Zaire: 1987. Clinical pediatrics. 1988 May; 27(5): 234-5.
118. El Shazly MK, Atta HY, Kishk NA. Poliomyelitis, measles and neonatal tetanus: a hospital based epidemiological study. The Journal of the Egyptian Public Health Association. 1997; 72(5-6): 527-48.
119. Commey JO, Richardson JE. Measles in Ghana--1973-1982. Annals of tropical paediatrics. 1984 Sep; 4(3): 189-94.
120. Bosu WK, Odoom S, Deiter P, Essel-Ahun M. Epidemiology of measles in the Central Region of Ghana: a five-year case review in three district hospitals. East African medical journal. 2003 Jun; 80(6): 312-7.

121. Aaby P, Martins C, Bale C, et al. Sex differences in the effect of vaccines on the risk of hospitalization due to measles in Guinea-bissau. The Pediatric infectious disease journal. 2010 Apr; 29(4): 324-8.
122. Rangkuti SM, Nazir N, Sutanto AH, Lubis A, Siregar H. Measles morbidity and mortality in the Department of Child Health, Dr. Pirngadi General Hospital, Medan, in 1973-1977. Paediatrica Indonesiana. 1980 Jul-Aug; 20(7-8): 139-44.
123. Lubis CP, Pasaribu S, Lubis MM. Morbidity and mortality of tetanus, diphtheria and morbilli (measles) cases (a 1982-1985 study at the Child Health Department, Dr. Pirngadi Hospital, Medan). The Journal of the Singapore Paediatric Society. 1987; 29(Suppl 1): 66-72.
124. Alwar AJ. The effect of protein energy malnutrition on morbidity and mortality due to measles at Kenyatta National Hospital, Nairobi (Kenya). East African medical journal. 1992 Aug; 69(8): 415-8.
125. Borus PK, Cumberland P, Sonoiya S, Kombich J, Tukei PM, Cutts FT. Measles trends and vaccine effectiveness in Nairobi, Kenya. East African medical journal. 2003 Jul; 80(7): 361-4.
126. Khoo A, Ho CK, Ong TK, Khairul A. Measles--an experience in Sandakan Hospital, Sabah, 1990. Singapore medical journal. 1994 Dec; 35(6): 595-8.
127. Boushab BM, Savadogo M, Sow MS, Dao S. [Epidemiological, clinical, and prognostic study of the measles in the Aioun regional hospital in Mauritania]. Medecine et sante tropicales. 2015 Apr-Jun; 25(2): 180-3. Aspects epidemiologiques, cliniques et pronostiques de la rougeole au centre hospitalier regional d'Aioun, Mauritanie.
128. Avila-Figueroa C, Navarrete-Navarro S, Martinez-Aguilar M, Ruiz-Gutierrez E, Santos JI. [Complications in children with measles]. Boletin medico del Hospital Infantil de Mexico. 1990 Jul; 47(7): 520-3. Complicaciones en ninos con sarampion.
129. Khin M, Win S, Aye SS. The impact of national measles immunization programme on measles admissions to the major children's hospital in Yangon. Tropical doctor. 1994 Jul; 24(3): 141-3.
130. Bamgboye EA, Familusi JB. Mortality pattern at a children's emergency ward, University College Hospital, Ibadan, Nigeria. African journal of medicine and medical sciences. 1990 Jun; 19(2): 127-32.
131. Adedoyin MA. The pattern of measles in Ilorin. West African journal of medicine. 1990 Apr-Jun; 9(2): 103-7.
132. Fagbule D, Orifunmishe F. Measles and childhood mortality in semi-urban Nigeria. African journal of medicine and medical sciences. 1988 Sep; 17(3): 181-5.
133. Ibia EO, Asindi AA. Measles in Nigerian children in Calabar during the era of expanded programme on immunization. Tropical and geographical medicine. 1990 Jul; 42(3): 226-32.
134. Ekanem EE, Ochigbo SO, Kwagtsule JU. Unprecedented decline in measles morbidity and mortality in Calabar, south-eastern Nigeria. Tropical doctor. 2000 Oct; 30(4): 207-9.
135. Fetuga MB, Jokanma OF, Ogunfowora OB, Abiodun R. A ten-year study of measles admissions in a Nigerian teaching hospital. Nigerian journal of clinical practice. 2007 Mar; 10(1): 41-6.
136. Lagunju IA, Orimadegun AE, Oyedemi DG. Measles in Ibadan: a continuous scourge. African journal of medicine and medical sciences. 2005 Dec; 34(4): 383-7.

137. Aurangzeb B, Nisar YB, Hazir T, Burki F, Hassan M. Clinical outcome in children hospitalized with complicated measles. Journal of the College of Physicians and Surgeons--Pakistan : JCPSP. 2005 Sep; 15(9): 547-51.
138. Anis ur R, Siddiqui TS, Idris M. Clinical outcome in measles patients hospitalized with complications. Journal of Ayub Medical College, Abbottabad : JAMC. 2008 Apr-Jun; 20(2): 14-6.
139. Mgone JM, Mgone CS, Duke T, Frank D, Yeka W. Control measures and the outcome of the measles epidemic of 1999 in the Eastern Highlands Province. Papua and New Guinea medical journal. 2000 Mar-Jun; 43(1-2): 91-7.
140. Benjamin AL, Dramoi V. Outbreak of measles in the National Capital District, Papua New Guinea in 2001. Papua and New Guinea medical journal. 2002 Sep-Dec; 45(3-4): 178-84.
141. Bronzwaer SL, de Groot CJ. [Risk factors for a complicated disease course in children with measles admitted to a Philippine university hospital]. Nederlands tijdschrift voor geneeskunde. 1997 Dec 20; 141(51): 2492-5. Risicofactoren voor gecompliceerd ziektebeloop bij kinderen opgenomen met mazelen in een Filippijns universitair ziekenhuis.
142. le Roux DM, le Roux SM, Nuttall JJ, Eley BS. South African measles outbreak 2009 - 2010 as experienced by a paediatric hospital. South African medical journal = Suid-Afrikaanse tydskrif vir geneeskunde. 2012 Sep; 102(9): 760-4.
143. Coetzee S, Morrow BM, Argent AC. Measles in a South African paediatric intensive care unit: again! Journal of paediatrics and child health. 2014 May; 50(5): 379-85.
144. AWahab MM, Ismail AM, Rais MA, Ishag YA. Measles in Equatoria region--south Sudan. Annals of tropical paediatrics. 1988 Mar; 8(1): 31-4.
145. Burgess W, Mduma B, Josephson GV. Measles in Mbeya, Tanzania--1981-1983. Journal of tropical pediatrics. 1986 Aug; 32(4): 148-53.
146. Oshitani H, Mpabalwani M, Kasolo F, et al. Measles infection in hospitalized children in Lusaka, Zambia. Annals of tropical paediatrics. 1995 Jun; 15(2): 167-72.
147. Uyirwoth GP. Measles in Mashonaland Central Province: Zimbabwe. East African medical journal. 1993 Jul; 70(7): 455-9.
148. Kambarami RA, Nathoo KJ, Nkrumah FK, Pirie DJ. Measles epidemic in Harare, Zimbabwe, despite high measles immunization coverage rates. Bulletin of the World Health Organization. 1991; 69(2): 213-9.
